# Facile modular synthesis of jasmonoyl-l-isoleucine analogs possessing a pyrazolidin-3-one core†

**DOI:** 10.1039/d3ra07887f

**Published:** 2024-01-25

**Authors:** Samuel Vizcaíno Páez, Diego Durango, Christian Jürgen Müller, Matthias Breuning, Wiston Quiñones Fletcher

**Affiliations:** a Química Orgánica de Productos Naturales, Universidad de Antioquia Medellín 050010 Antioquia Colombia wiston.quinones@udea.edu.co; b Química de los Productos Naturales y los Alimentos, Universidad Nacional de Colombia Medellín 050034 Antioquia Colombia; c Department of Chemistry, University of Bayreuth, Universitätsstraße 30 95447 Bayreuth Germany matthias.breuning@uni-bayreuth.de

## Abstract

A short and flexible route to pyrazolidin-3-one analogs of the phytohormone (+)-7-iso-jasmonoyl-l-isoleucine is presented. The compounds were assembled from four basic building blocks, namely a pyrazolidin-3-one core, alkyl chain, linker and amino ester or acid. The efficacy of this approach was demonstrated in the synthesis of 11 analogs with variations in all parts of the molecule.

## Abstract 2

Several pyrazolidine-3-one analogs of (+)-7-iso-jasmonoyl-l-isoleucine, the main phytohormone involved in plant responses to biotic or abiotic stress, were prepared from 4 different building blocks in just a few steps.

## Graphical abstract

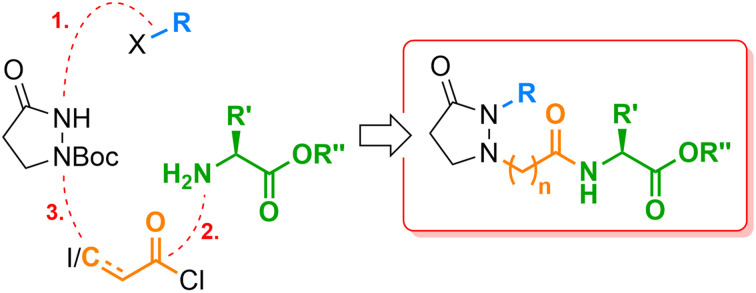

## Introduction

**Fig. 1:**
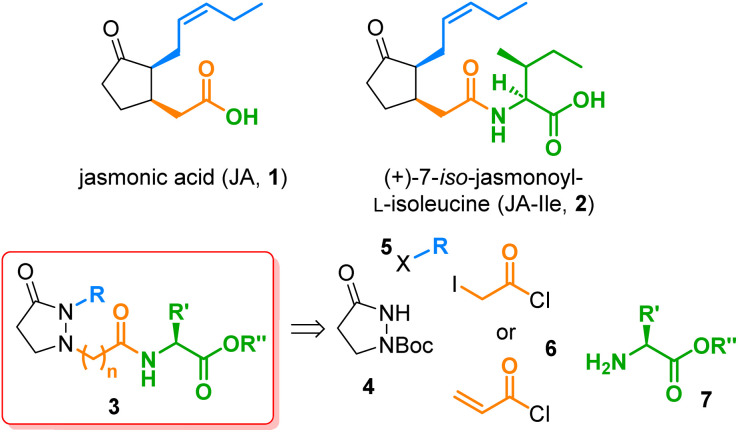
The phytohormones jasmonic acid (1) and (+)-7-iso-jasmonoyl-l-isoleucine (JA-Ile, 2), the new pyrazolidin-3-one based JA-Ile analogs 3, and their four building blocks 4–7.

The jasmonates (JAs), which constitute a family of more than 30 secondary metabolites derived from jasmonic acid (1) (Fig. 1), fulfill multiple physiological roles throughout the plant kingdom.^1^ The most remarkable molecule from this group is (+)-7-iso-jasmonoyl-l-isoleucine (JA-Ile, 2), identified as the cornerstone for plant responses to biotic stress.^2^ Other jasmonate-induced physiological responses observed in higher plants include inhibition of cytokinin-induced growth,^3^ stimulation of ethylene production, induction of proteinase inhibitor II,^4,5^ tuber inducing,^6^ accumulation of secondary metabolites,^7^ tendril coiling,^8^ odor production,^9,10^ JIP-23 gene expression,^11^ phytoalexin production,^12^ and stomatal opening.^13^ The importance of these jasmonate-induced responses has triggered the interest in developing synthetic routes to JA analogs.^14^ The prepared analogs permitted structure activity relationship (SAR) studies through which the main structural features required to keep or to potentiate such activities were identified. SAR studies have been done on activities in plants such as tendril coiling^15^ or secondary metabolites accumulation,^16^ and even in humans, such as anti-inflammatory,^17^ neoplastic,^18^ or anticancer,^19^ because of the structural similarity of these compounds to prostaglandins in mammals. Notwithstanding, all existing routes to JAs analogs are mainly linear, including *de novo* syntheses^20–22^ and partial syntheses starting from commercially available methyl jasmonate or *cis*-jasmone.^23–25^ In an interesting approach to prostaglandin E_2_ analogs, the cyclopentanone moiety was replaced by a pyrazolidin-3-one, yielding several potent and selective *in vitro* EP_2_ and EP_4_ receptor agonists.^26^

A similar substitution in the field of JA-Ile (2), *viz.* the replacement of the chiral cyclopentanone core by a simple, achiral pyrazolidine-3-one, would lead to analogs of type 3, which should be by far more easily accessible since all stereocenters are omitted and the nitrogen atoms provide versatile attachment points for side chains. Consequently, a quick preparation of a structurally broad library of JA-Ile derivatives, as required for in-depth SAR studies, would be possible. In this work, we realized this idea and synthesized a couple of JA-Ile analogs 3. We developed a convergent route based on four different building blocks, the pyrazolidin-3-one core 4, the alkyl side chain 5, the acid linker 6, and the terminal amino acid 7. This strategy is highly efficient and permits access to a wide range of structural modifications, because all building blocks can be varied independently and prior to the final coupling stage.

## Results and discussion

The syntheses of the core building blocks 10 commenced with methyl acrylate (8), which was cyclized with hydrazine hydrate. After *N*-Boc protection of the amino function, which is required in order to direct the following alkylation to the less nucleophilic amide nitrogen, the pyrazolidin-3-one 4 was obtained in good 72% yield. Deprotonation of the amide with NaH in DMF at 0 °C and subsequent electrophilic quench with (*Z*)-pent-2-en-1-yl methanesulfonate (5a), 1-bromopentane (5b), 1-bromohexane (5c), and 1-bromobutane (5d), respectively, introduced the first variable substituent R and provided the protected pyrazolidin-3-ones 9a–d in reasonable yields (55–63%). As side products, minor quantities (<15%) of the corresponding *O*-alkylated imidates were observed, which was characterized in the case of that formed together with 9b. We also explored an alternative route to 9 based on an inversed reaction sequence, namely the alkylation of *tert*-butyl carbazate followed by cyclization with 3-chloropropionyl chloride or ethyl acrylate (8),^26^ which, however, was met with low success and provided only traces of 9a. Final deprotection of 9a–d with TFA afforded the core building blocks 10a–d in excellent yield (>97%) and overall just four steps from methyl acrylate (Table 1).

**Table 1:** Synthesis of the pyrazolidine-3-one cores 10^a^

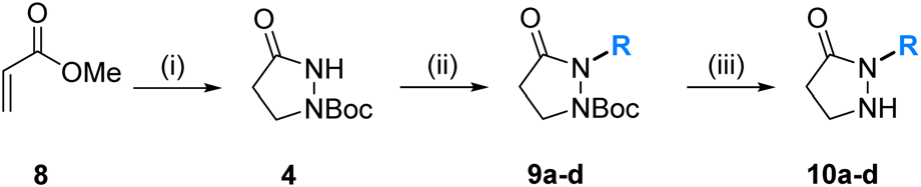			
Entry	R	Cmpd^b^ (Yield [%])	Cmpd^b^ (Yield [%])
1	(*Z*)-2-Pentenyl	9a (55)	10a (99)
2	*n*Pent	9b (67)	10b (99)
3	*n*Hex	9c (57)	10c (99)
4	*n*Bu	9d (63)	10d (97)

a Reaction conditions: (i) 1. H_2_NNH_2_·H_2_O, mol sieves 3 Å, EtOH, 0 °C to reflux, 22.5 h; 2. Boc_2_O, MeOH, rt, 5 h, 72%; (ii) NaH, DMF, 0 °C, 30 min, then R–X (5a–d), rt, 18 h; (iii) TFA, CHCl_3_, rt, 18 h.

b Isolated yields.

The side chain connected to the amino group of 3 consists of an acid linker and an amino acid, which both were independently varied. In a first set, the methyl esters of l-isoleucine (7a), l-leucine (7b), l-valine (7c), and l-alanine (7d), which all possess aliphatic and hydrophobic side chains as found in JA-Ile (2), were chosen as models for the amino acid part. Note that the esters are eligible bio-precursors for the corresponding amino acids in the final products because saponification will readily occur in a living environment.^2^ According to a known procedure,^27,28^7a–d were treated with chloroacetyl chloride (11) and NEt_3_, which introduced the ‘natural’ two-carbon linker of JA-Ile (2) and furnished the known chlorides 12aa–ad in excellent yields above 90%. It should be stressed that, under slightly modified conditions,^29^ this procedure is also applicable to unprotected amino acids. The chloride 12ae was obtained in good 80% yield from 11 and two equivalents of l-isoleucine (7e) in MeCN at −20 °C. Cl/I-exchange in 12aa–ae under Finkelstein conditions, in order to improve the quality of the leaving group for the final coupling, delivered the desired iodides 6aa–6ae in high 74–94% yield. Note that compounds of type 6ae and 12ae, which possess a free acid group and a leaving group like chloride or iodine, are prone to undergo intramolecular cyclization to morpholin-2,5-diones.^30^ In our case, however, only traces of such by-products were observed. Finally, a precursor for a side chain with a longer three-carbon linker was prepared, too. Treatment of acryloyl chloride (13) with l-isoleucine methyl ester (7a) furnished the enamide 6ba in 77% yield (Table 2).

**Table 2:** Synthesis of the amino acid side chains 6^a^

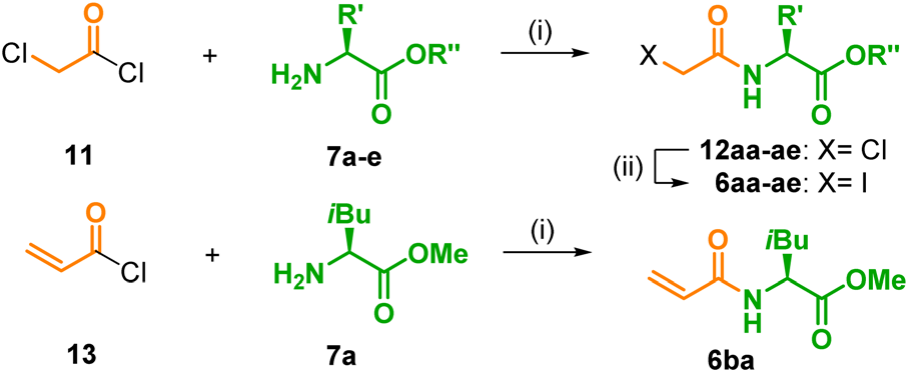				
Entry	Cmpd	R′^b^	R′′	Yield^c^ [%]
1	6aa	sBu (Ile)	Me	80
2	6ab	iBu (Leu)	Me	94
3	6ac	iPr (Val)	Me	74
4	6ad	Me (Ala)	Me	77
5	6ae	sBu (Ile)	H	89
6	6ba	sBu (Ile)	Me	77

a Reaction conditions: (i) for 12aa–ad and 6ba: Et_3_N, CHCl_3_, 0 °C, 1 h, 62–96%.^31,32^ for 12ae: Et_3_N, MeCN, −20 °C, 48 h, 92%; (ii) KI, acetone, rt, 24 h.

b In brackets: corresponding l-amino acid.

c Isolated yields.

The final coupling step between the pyrazolidin-3-one cores 10a–d and the side chains 6aa–ad occurred smoothly under S_N_2-conditions (Cs_2_CO_3_, DMF) at room temperature, giving the desired JA-Ile analogs 3 in good to high yields (48–83%). Mol sieves 4 Å was added to remove traces of water, as formed by the carbonate, to prevent any yield-lowering solvolysis of the iodide. An aza-Michael addition was intended for the linkage of the α,β-unsaturated amide 6ba to the core 10a. Under standard conditions with MeOH as the solvent, only an oxo-Michael addition of MeOH was observed. We therefore changed to more forcing conditions in a polar, but low-nucleophilic solvent (TFE/H_2_O 1 : 1, 100 °C),^31,32^ under which the desired aza-Michael product 3aba was formed in acceptable 58% yield (Table 3).

**Table 3:** Synthesis of the JA-Ile analogs 3^a^

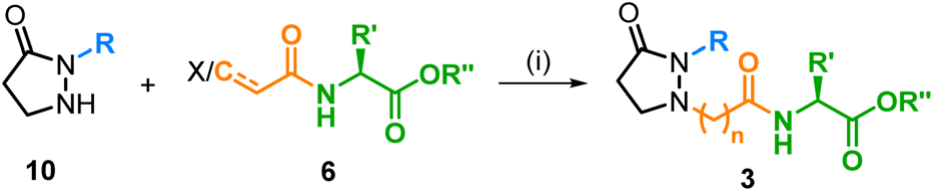						
Entry	Cmpd	R	*n*	R′	R′′	Yield^b^ [%]
1	3aaa	(*Z*)-2-Pentenyl	1	sBu	Me	70
2	3aab	(*Z*)-2-Pentenyl	1	iBu	Me	63
3	3aac	(*Z*)-2-Pentenyl	1	iPr	Me	77
4	3aad	(*Z*)-2-Pentenyl	1	Me	Me	48
5	3aae	(*Z*)-2-Pentenyl	1	sBu	H	77
6	3baa	*n*Pent	1	sBu	Me	83
7	3caa	*n*Hex	1	sBu	Me	66
8	3daa	*n*Bu	1	sBu	Me	74
9	3aba	(*Z*)-2-Pentenyl	2	sBu	Me	58
10	3bab	*n*Pent	1	iBu	Me	52

a Reaction conditions: (i) for 6aa–ad: Cs_2_CO_3_, DMF, mol sieves 4 Å, rt, 16–48 h; for 6ba: TFE/H_2_O 1 : 1, 100 °C, 24 h.

b Isolated yields.

## Conclusions

A modular and convergent strategy for the synthesis of pyrazolidin-3-one analogs 3 of the key phytohormone involved in the plant chemical responses to biotic or abiotic stress JA-Ile has been developed. The four basic building blocks, the achiral pyrazolidin-3-one core 4, the alkyl side chain 5, the acid linker 6, and the terminal amino acid ester 7, can be independently varied and combined without restrictions, thus permitting a facile access to a structurally broad array of compounds in just a few steps. The high potential and efficacy of this approach was proven in the synthesis of eleven different JA-Ile analogs 3, which include variations at all possible positions. Any epimerization in the amino acid side chain was excluded. By using this approach, large libraries of JA-Ile analogs 3 are now easily accessible, which can be used for in-depth SAR studies on JA (1) or JA-Ile (2) triggered biological processes. Investigations in this direction are in progress.

## Experimental

### General

All reactions with moisture-sensitive reagents were carried out under an Ar atmosphere in anhydrous solvents, which were prepared using standard procedures. Commercially available reagents were used as received. Reaction monitoring was done by thin-layer chromatography (TLC) on precoated silica gel plates (Merck TLC Silica gel 60 F_254_). Spots were visualized by UV light (254 nm or 366 nm) or by staining with aqueous KMnO_4_, vanillin, or phosphomolybdic acid (PMA). Silica gel (40–63 μm) was used for column chromatography. Melting points were measured on a Büchi M-565 melting point apparatus and are uncorrected. Optical rotations were recorded through 5 continuous repetitions on a Jasco P-2000 polarimeter (1 dm cell) at 589 nm and concentrations close to 1.0% in chloroform or methanol. Infrared spectra were recorded on a PerkinElmer Spectrum 100 FT-IR spectrometer. NMR spectra were measured on Bruker Avance III HD 300 and 500 instruments and calibrated using the residual non-deuterated solvent as an internal reference. High resolution mass spectrometry (HRMS) was performed on a ThermoFischer Scientific Q-Extractive (Orbitrap) mass spectrometer using electrospray ionization (ESI). Compounds 12aa–ad were synthesized in 92–96% yield according to literature.^27,28^

### Synthesis

#### *tert*-Butyl 3-oxopyrazolidine-1-carboxylate (4)

Powdered mol sieves 3 Å (40 mg) was added to a solution of ethanol (8.0 mL) and hydrazine hydrate (1.00 mL, 20.0 mmol). Methyl acrylate (2.00 mL, 22.1 mmol) was added dropwise at 0 °C and, after 90 min at 0 °C, the mixture was refluxed under an Ar atmosphere for 21 h. The solvent was removed under reduced pressure and the residue was dissolved in methanol (20.0 mL). Boc anhydride (4.80 g, 22.0 mmol) was added and the mixture was stirred for 5 h at rt. After addition of aqueous HCl (1 N, 40 mL), the reaction mixture was extracted with CH_2_Cl_2_ (3 × 40 mL), and the combined organic layers were dried over MgSO_4_. Evaporation of the solvent under reduced pressure and column chromatography (silica gel, CH_2_Cl_2_/MeOH 98 : 2 → 90 : 10) delivered product 4 (2.70 g, 14.4 mmol, 72%) as white needles. Mp. 107–110 °C. FTIR (ATR): *ν̃*_max_/cm^−1^ 3301 (NH), 2978, 2933, 1686 (C=O).·^1^H NMR (500 MHz, CDCl_3_): *δ* (ppm) 8.56 (s, 1H), 3.95 (t, *J* = 8.6 Hz, 2H), 2.67 (t, *J* = 8.6 Hz, 2H), 1.49 (s, 9H). ^13^C NMR (125 MHz, CDCl_3_): *δ* (ppm) 170.72 (C=O), 153.78 (C=O), 82.75 (C_q_), 44.82 (CH_2_), 31.09 (CH_2_), 28.36 (CH_3_).

#### *tert*-Butyl 3-oxo-2-[(2*Z*)-pent-2-en-1-yl]pyrazolidine-1-carboxylate (9a)

Under an Ar atmosphere, a 1.0 M solution of 4 (88.0 mg, 0.473 mmol) in anhydrous DMF (88.0 μL) was cooled to 0 °C. NaH (23.0 mg, 0.567 mmol) was added and the solution was stirred for 30 min. (2*Z*)-Pent-2-en-1-yl methanesulfonate^21^ (2a, 90.0 mg, 0.520 mmol), dissolved in anhydrous DMF (1.0 mL), was added dropwise. The mixture was allowed to reach rt and stirred for 18 h. Brine (5 mL) was added and the mixture was extracted with EtOAc (3 × 15 mL). The organic layers were combined, dried over Na_2_SO_4_, and concentrated under reduced pressure. Column chromatography (silica gel, hexane/EtOAc 85 : 15) provided the 9a (66.0 mg, 0.263 mmol, 55%) as a colorless oil. FTIR (ATR): *ν̃*_max_/cm^−1^ 3009 (C=CH), 2973, 2935, 2875, 1704 (C=O), 1640 (C=C), 1314, 1153. ^1^H NMR (500 MHz, CDCl_3_): *δ* (ppm) 5.66–5.58 (m, 1H), 5.36–5.28 (m, 1H), 4.34 (d, *J* = 7.1 Hz, 2H), 3.94 (t, *J* = 7.7 Hz, 2H), 2.54 (t, *J* = 7.7 Hz, 2H), 2.11 (pd, *J* = 7.5, 1.2 Hz, 2H), 1.50 (s, 9H), 0.97 (t, *J* = 7.5 Hz, 3H). ^13^C NMR (125 MHz, CDCl_3_): *δ* (ppm) 172.02 (C=O), 156.93 (C=O), 137.16 (=CH), 122.01 (=CH), 82.77 (C_q_), 47.80 (CH_2_), 42.13 (CH_2_), 31.44 (CH_2_), 28.27 (CH_3_), 20.86 (CH_2_), 14.39 (CH_3_). HRMS (+ESI): *m*/*z* calcd for C_13_H_23_N_2_O_3_^+^ [M + H]^+^: 255.1703; found: 255.1698.

#### *tert*-Butyl 3-oxo-2-pentylpyrazolidine-1-carboxylate (9b)

Compound 9b was prepared from 4 (466 mg, 2.50 mmol) and 1-bromopentane (2b, 475 μL, 3.75 mmol), as described for 9a. Yield: 428 mg (1.67 mmol, 67%), colorless oil. FTIR (ATR): *ν̃*_max_/cm^−1^ 2958, 2933, 2874, 1702 (C=O), 1315, 1155. ^1^H NMR (500 MHz, CDCl_3_): *δ* (ppm) 3.95 (t, *J* = 7.7 Hz, 2H), 3.69 (t, *J* = 7.2 Hz, 2H), 2.55 (t, *J* = 7.7 Hz, 2H), 1.57 (p, *J* = 7.4 Hz, 2H), 1.50 (s, 9H), 1.38–1.28 (m, 2H), 1.28–1.20 (m, 2H), 0.88 (t, *J* = 7.2 Hz, 3H). ^13^C NMR (125 MHz, CDCl_3_): *δ* (ppm) 171.57 (C=O), 157.05 (C=O), 82.83 (C_q_), 47.63 (CH_2_), 45.18 (CH_2_), 31.65 (CH_2_), 28.99 (CH_2_), 28.30 (CH_3_), 26.16 (CH_2_), 22.44 (CH_2_), 14.11 (CH_3_). HRMS (+ESI): *m*/*z* calcd for C_13_H_25_N_2_O_3_^+^ [M + H]^+^: 257.1860; found: 257.1855.

#### *tert*-Butyl 3-oxo-2-hexylpyrazolidine-1-carboxylate (9c)

Compound 9c was prepared from 4 (466 mg, 2.50 mmol) and 1-bromohexane (2c, 531 μL, 3.76 mmol), as described for 9a. Yield: 384 mg (1.42 mmol, 57%), colorless oil. FTIR (ATR): *ν̃*_max_/cm^−1^ 2959, 2931, 2860, 1703 (C=O), 1314, 1155. ^1^H NMR (500 MHz, CDCl_3_): *δ* (ppm) 3.95 (t, *J* = 7.7 Hz, 2H), 3.69 (t, *J* = 7.2 Hz, 2H), 2.55 (t, *J* = 7.7 Hz, 2H), 1.56 (p, *J* = 7.2 Hz, 2H), 1.50 (s, 9H), 1.33–1.22 (m, 6H), 0.87 (t, *J* = 6.9 Hz, 3H). ^13^C NMR (125 MHz, CDCl_3_): *δ* (ppm) 171.58 (C=O), 157.06 (C=O), 82.85 (C_q_), 47.64 (CH_2_), 45.22 (CH_2_), 31.66 (CH_2_), 31.55 (CH_2_), 28.31 (CH_3_), 26.51 (CH_2_), 26.44 (CH_2_), 22.65 (CH_2_), 14.14 (CH_3_). HRMS (+ESI): *m*/*z* calcd for C_14_H_27_N_2_O_3_^+^ [M + H]^+^: 271.2016; found: 271.2010.

#### *tert*-Butyl 3-oxo-2-butylpyrazolidine-1-carboxylate (9d)

Compound 9d was prepared from 4 (466 mg, 2.50 mmol) and 1-bromobutane (2d, 412 μL, 3.76 mmol), as described for 9a. Yield: 383 mg (1.58 mmol, 63%), colorless oil. FTIR (ATR): *ν̃*_max_/cm^−1^ 2960, 2934, 2875, 1701 (C=O), 1316, 1155. ^1^H NMR (500 MHz, CDCl_3_): *δ* (ppm) 3.94 (t, *J* = 7.8 Hz, 2H), 3.68 (t, *J* = 7.2 Hz, 2H), 2.53 (t, *J* = 7.8 Hz, 2H), 1.54 (p, 7.4 Hz, 2H), 1.48 (s, 9H), 1.27 (sext, *J* = 7.5 Hz, 2H), 0.90 (t, *J* = 7.4 Hz, 3H). ^13^C NMR (125 MHz, CDCl_3_): *δ* (ppm) 171.54 (C=O), 157.01 (C=O), 82.79 (C_q_), 47.59 (CH_2_), 44.85 (CH_2_), 31.60 (CH_2_), 28.52 (CH_3_), 28.26 (CH_2_), 20.02 (CH_2_), 13.83 (CH_3_). HRMS (+ESI): *m*/*z* calcd for C_12_H_23_N_2_O_3_^+^ [M + H]^+^: 243.1703; found: 243.1699.

#### 2-[(2*Z*)-Pent-2-en-1-yl]pyrazolidin-3-one (10a)

Under an Ar atmosphere, TFA (3.5 mL, 45.4 mmol) was added dropwise to a 0.35 M solution of 9a (1.14 g, 4.49 mmol) in HCCl_3_ (13 mL). After 24 h, the mixture was concentrated and crude 10a was obtained by column chromatography (silica gel, DCM/MeOH/NH_3_ (aq, 25%) 97.1 : 2.6 : 0.3). Traces of TFA were removed by treatment with 3 M KOH (1.0 mL), followed by extraction with EtOAc (3 × 10 mL). Evaporation of the solvent delivered analytically pure amine 10a (688 mg, 4.46 mmol, 99%) as an orange oil. FTIR (ATR): *ν̃*_max_/cm^−1^ 3218 (NH), 3020 (C=CH), 2966, 2936, 2878, 1666 (C=O). ^1^H NMR (500 MHz, CDCl_3_): *δ* (ppm) 5.68–5.61 (m, 1H), 5.38–5.31 (m, 1H), 4.37 (s, 1H, N–H), 4.08 (d, *J* = 7.0 Hz, 2H), 3.37 (t, *J* = 7.8 Hz, 2H), 2.54 (t, *J* = 7.7 Hz, 2H), 2.15 (pd, *J* = 7.5, 1.3 Hz, 2H), 0.99 (t, *J* = 7.5 Hz, 3H). ^13^C NMR (125 MHz, CDCl_3_): *δ* (ppm) 171.88 (C=O), 137.17 (=CH), 122.17 (=CH), 43.83 (CH_2_), 40.67 (CH_2_), 33.35 (CH_2_), 20.80 (CH_2_), 14.27 (CH_3_). HRMS (+ESI): *m*/*z* calcd for C_8_H_15_N_2_O^+^ [M + H]^+^: 155.1179; found: 155.1175.

#### 2-Pentylpyrazolidin-3-one (10b)

Compound 10b was prepared from 9b (400 mg, 1.56 mmol), as described for 10a. Yield: 244 mg (1.56 mmol, 99%), orange oil. FTIR (ATR): *ν̃*_max_/cm^−1^ 3216 (NH), 2957, 2930, 2872, 1663 (C=O). ^1^H NMR (500 MHz, CDCl_3_): *δ* (ppm) 4.31 (s, 1H, N–H), 3.41 (t, *J* = 7.2 Hz, 2H), 3.36 (t, *J* = 7.7 Hz, 2H), 2.55 (t, *J* = 7.6 Hz, 2H), 1.58 (p, *J* = 7.4 Hz, 2H), 1.38–1.23 (m, 4H), 0.89 (t, *J* = 7.1 Hz, 3H). ^13^C NMR (125 MHz, CDCl_3_): *δ* (ppm) 172.27 (C=O), 44.18 (CH_2_), 44.12 (CH_2_), 33.67 (CH_2_), 29.07 (CH_2_), 27.06 (CH_2_), 22.46 (CH_2_), 14.13 (CH_3_). HRMS (+ESI): *m*/*z* calcd for C_8_H_17_N_2_O^+^ [M + H]^+^: 157.1335; found: 157.1330.

#### 2-Hexylpyrazolidin-3-one (10c)

Compound 10c was prepared from 9c (355 mg, 1.31 mmol), as described for 10a. Yield: 223 mg (1.31 mmol, 99%), orange oil. FTIR (ATR): *ν̃*_max_/cm^−1^ 3215 (NH), 2959, 2929, 2858, 1663 (C=O). ^1^H NMR (500 MHz, CDCl_3_): *δ* (ppm) 4.35 (s, 1H, N–H), 3.41 (t, *J* = 7.2 Hz, 2H), 3.36 (t, *J* = 7.7 Hz, 2H), 2.54 (t, *J* = 7.5 Hz, 2H), 1.57 (p, *J* = 7.1 Hz, 2H), 1.33–1.25 (m, 6H), 0.87 (t, *J* = 6.7 Hz, 3H). ^13^C NMR (125 MHz, CDCl_3_): *δ* (ppm) 172.26 (C=O), 44.18 (CH_2_), 44.15 (CH_2_), 33.67 (CH_2_), 31.58 (CH_2_), 27.33 (CH_2_), 26.59 (CH_2_), 22.67 (CH_2_), 14.15 (CH_3_). HRMS (+ESI): *m*/*z* calcd for C_9_H_19_N_2_O^+^ [M + H]^+^: 171.1492; found: 171.1487.

#### 2-Butylpyrazolidin-3-one (10d)

Compound 10d was prepared from 9d (348 mg, 1.43 mmol), as described for 10a. Yield: 196 mg (1.39 mg, 97%), orange oil. FTIR (ATR): *ν̃*_max_/cm^−1^ 3216 (NH), 2960, 2934, 2875, 1662 (C=O). ^1^H NMR (500 MHz, CDCl_3_): *δ* (ppm) 4.36 (s, 1H, N–H), 3.41 (t, *J* = 7.2 Hz, 2H), 3.35 (t, *J* = 7.7 Hz, 2H), 2.53 (t, *J* = 7.6 Hz, 2H), 1.55 (p, *J* = 7.4 Hz, 2H), 1.32 (sext, *J* = 7.5 Hz, 2H), 0.92 (t, *J* = 7.4 Hz, 3H). ^13^C NMR (125 MHz, CDCl_3_): *δ* (ppm) 172.27 (C=O), 44.16 (CH_2_), 43.82 (CH_2_), 33.64 (CH_2_), 29.40 (CH_2_), 20.11 (CH_2_), 13.84 (CH_2_). HRMS (+ESI): *m*/*z* calcd for C_7_H_15_N_2_O^+^ [M + H]^+^: 143.1179; found: 143.1175.

#### Preparation of the l-*N*-chloroacetyl amino acid methyl esters 12aa–ad

The *N*-chloroacetylations of the amino esters 7a–d were performed according to the procedure described by Han and Gong.^27^ The spectroscopic data of the products 12aa–ad were identical to those reported by White *et al.*^28^

#### (2*S*,3*S*)-2-[(Chloroacetyl)amino]-3-methylpentanoic acid (12ae)

l-Isoleucine (7e, 265 mg, 2.0 mmol) was suspended in MeCN (2.0 mL) and stirred at −20 °C by 30 min. Then chloroacetyl chloride (81.0 μL, 1.00 mmol) was added dropwise and the mixture kept under stirring at this temperature. After 48 h it was filtered through glass wool and rinsed with cooled MeCN (2.0 mL). Concentration provided 12ae (191 mg, 0.918 mmol, 92%) as white crystals. Mp. 62–65 °C. [*α*]^24^_D_ + 40.8 (c 1.0, HCCl_3_). FTIR (ATR): *ν̃*_max_/cm^−1^ 3500–2500 (COOH), 3364 (NH), 2964, 2925, 2878, 1709 (C=O), 1622 (C=O), 1533 (NH), 1235. ^1^H NMR (500 MHz, CDCl_3_): *δ* (ppm) 7.06 (d, *J* = 8.4 Hz, 1H, N–H), 4.63 (dd, *J* = 8.6, 4.6 Hz, 1H), 4.12 (s, 2H, CH_2_Cl), 2.07–1.98 (m, 2H), 1.57–1.47 (m, 1H), 1.33–1.20 (m, 1H), 0.99 (d, *J* = 6.8 Hz, 3H), 0.97 (t, *J* = 7.3 Hz, 3H). ^13^C NMR (125 MHz, CDCl_3_): *δ* (ppm) 175.85 (C=O), 166.36 (C=O), 56.81 (CH), 42.67 (CH_2_), 37.74 (CH), 25.14 (CH_2_), 15.58 (CH_3_), 11.73 (CH_3_). HRMS (+ESI): *m*/*z* calcd for C_8_H_15_NClO_3_^+^ [M + H]^+^: 210.0735; found: 208.0734.

#### Methyl (2*S*,3*S*)-2-[(iodoacetyl)amino]-3-methylpentanoate (6aa)

Under an Ar atmosphere, a 1.0 M solution of 12aa (2.80 g, 12.4 mmol) in anhydrous acetone (12.4 mL) was prepared. KI (4.13 g, 24.9 mmol, 2.0 eq.) was added and the reaction mixture was stirred for 24 h in the dark. The supernatant was filtered through Celite® 545, the filter cake was rinsed with acetone (5.0 mL), and the filtrate was concentrated under vacuum. The residue was treated with aq. Na_2_S_2_O_3_ (10%, 10 mL) and extracted with EtOAc (3 × 25 mL). The combined organic layers were concentrated to give analytically pure 6aa (3.11 g, 9.93 mmol, 80%) as an orange solid. Mp. 40–43 °C. [*α*]^27^_D_ + 20.0 (c 1.0, HCCl_3_). FTIR (ATR): *ν̃*_max_/cm^−1^ 3317 (NH), 2969, 2936, 2879, 1736 (C=O), 1644 (C=O), 1536 (NH). ^1^H NMR (500 MHz, CDCl_3_): *δ* (ppm) 6.49 (d, *J* = 7.6 Hz, 1H, N–H), 4.57 (dd, *J* = 8.6, 4.8 Hz, 1H), 3.75 (s, 3H, OCH_3_), 3.75 (d, *J* = 11.5 Hz, 1H), 3.70 (d, *J* = 11.5 Hz, 1H), 1.99–1.90 (m, 1H), 1.50–1.40 (m, 1H), 1.27–1.15 (m, 1H), 0.94 (t, *J* = 7.4 Hz, 3H), 0.93 (d, *J* = 6.9 Hz, 3H). ^13^C NMR (125 MHz, CDCl_3_): *δ* (ppm) 172.19 (C=O), 166.81 (C=O), 57.23 (CH), 52.43 (OCH_3_), 38.18 (CH), 25.22 (CH_2_), 15.52 (CH_3_), 11.73 (CH_3_), −0.90 (ICH_2_). HRMS (+ESI): *m*/*z* calcd for C_9_H_17_NIO_3_^+^ [M + H]^+^: 314.0248; found: 314.0243.

#### Methyl (2*S*)-2-[(iodoacetyl)amino]-4-methylpentanoate (6ab)

Compound 6ab was prepared from 12ab (315 mg, 1.42 mmol), as described for 6aa. Yield: 419 mg (1.34 mmol, 94%), orange oil. [*α*]^27^_D_ − 2.6 (c 1.0, HCCl_3_). FTIR (ATR): *ν̃*_max_/cm^−1^ 3287 (NH), 2956, 2871, 1742 (C=O), 1648 (C=O), 1539 (NH). ^1^H NMR (500 MHz, CDCl_3_): *δ* (ppm) 6.37 (d, *J* = 7.3 Hz, 1H, N–H), 4.61 (td, *J* = 8.6, 4.9 Hz, 1H), 3.75 (s, 3H, OCH_3_), 3.74 (d, *J* = 11.1 Hz, 1H, H–CHI), 3.70 (d, *J* = 11.5 Hz, 1H, H–CHI), 1.73–1.63 (m, 2H), 1.63–1.53 (m, 1H), 0.95 (d, *J* = 6.1 Hz, 3H), 0.95 (d, *J* = 6.2 Hz, 3H). ^13^C NMR (125 MHz, CDCl_3_): *δ* (ppm) 173.26 (C=O), 166.92 (C=O), 57.62 (CH), 51.59 (OCH_3_), 41.63 (CH_2_), 24.97 (CH), 22.94 (CH_3_), 22.10 (CH_3_), −1.13 (ICH_2_). HRMS (+ESI): *m*/*z* calcd for C_9_H_17_NIO_3_^+^ [M + H]^+^: 314.0248; found: 314.0243.

#### Methyl (2*S*)-2-[(iodoacetyl)amino]-3-methylbutanoate (6ac)

Compound 6ac was prepared from 12ac (72.0 mg, 0.339 mmol), as described for 6aa. Yield: 75.0 mg (0.251 mmol, 74%), orange solid. Mp. 47–50 °C. [*α*]^27^_D_ + 13.0 (c 1.0, HCCl_3_). FTIR (ATR): *ν̃*_max_/cm^−1^ 3279 (NH), 2965, 2902, 2875, 1723 (C=O), 1640 (C=O), 1548 (NH). ^1^H NMR (500 MHz, CDCl_3_): *δ* (ppm) 6.49 (d, *J* = 7.4 Hz, 1H, N–H), 4.53 (dd, *J* = 8.8, 4.8 Hz, 1H), 3.76 (d, *J* = 11.4 Hz, 1H, H–CHI), 3.76 (s, 3H, OCH_3_), 3.71 (d, *J* = 11.4 Hz, 1H, H–CHI), 2.22 (septd, *J* = 6.9, 4.8 Hz, 1H), 0.96 (d, *J* = 6.9 Hz, 3H), 0.93 (d, *J* = 6.9 Hz, 3H). ^13^C NMR (125 MHz, CDCl_3_): *δ* (ppm) 172.23 (C=O), 167.05 (C=O), 57.82 (CH), 52.49 (OCH_3_), 31.65 (CH), 19.01 (CH_3_), 17.78 (CH_3_), −0.98 (ICH_2_). HRMS (+ESI): *m*/*z* calcd for C_8_H_15_NIO_3_^+^ [M + H]^+^: 300.0091; found: 300.0086.

#### Methyl (2*S*)-2-[(iodoacetyl)amino]propanoate (6ad)

Compound 6ad was prepared from 12ad (217 mg, 1.21 mmol), as described for 6aa. Yield: 253 mg (0.934 mmol, 77%), yellow solid. Mp. 55–57 °C. [*α*]^27^_D_ + 7.4 (c 1.0, HCCl_3_). FTIR (ATR): *ν̃*_max_/cm^−1^ 3285 (NH), 2966, 2952, 2928, 1722 (C=O), 1644 (C=O), 1542 (NH). ^1^H NMR (500 MHz, CDCl_3_): *δ* (ppm) 6.58 (s, 1H, N–H), 4.57 (p, *J* = 7.2 Hz, 1H), 3.77 (s, 3H, OCH_3_), 3.71 (s, 2H, CH_2_I), 1.43 (d, *J* = 7.2 Hz, 3H). ^13^C NMR (125 MHz, CDCl_3_): *δ* (ppm) 173.20 (C=O), 166.55 (C=O), 52.80 (OCH_3_), 48.98 (CH), 18.27 (CH_3_), −1.09 (ICH_2_). HRMS (+ESI): *m*/*z* calcd for C_6_H_11_NIO_3_^+^ [M + H]^+^: 271.9778; found: 271.9776.

#### (2*S*,3*S*)-2-[(Iodoacetyl)amino]-3-methylpentanoic acid (6ae)

Compound 6ae was prepared from 12ae (96.0 mg, 0.460 mmol), as described for 6aa. Yield 122 mg (0.408 mmol, 89%), pale yellow solid. Mp. 143–145 °C. [*α*]^26^_D_ + 0.8 (c 1.0, MeOH). FTIR (ATR): *ν̃*_max_/cm^−1^ 3292 (NH), 3200–2700 (COOH), 2968, 2957, 2877, 1704 (C=O), 1623 (C=O), 1557 (NH), 1255. ^1^H NMR (500 MHz, CD_3_OD): *δ* (ppm) 4.33 (d, *J* = 5.8 Hz, 1H), 3.86 (d, *J* = 9.8 Hz, 1H, H–CHI), 3.75 (d, *J* = 9.8 Hz, 1H, H–CHI), 1.99–1.89 (m, 1H), 1.58–1.48 (m, 1H), 1.33–1.22 (m, 1H), 0.97 (d, *J* = 6.9 Hz, 3H), 0.93 (t, *J* = 7.4 Hz, 3H). ^13^C NMR (125 MHz, CD_3_OD): *δ* (ppm) 174.47 (C=O), 171.38 (C=O), 58.57 (CH), 38.35 (CH), 26.03 (CH_2_), 16.01 (CH_3_), 11.76 (CH_3_), −2.05 (ICH_2_). HRMS (+ESI): *m*/*z* calcd for C_8_H_15_NIO_3_^+^ [M + H]^+^: 300.0091; found: 300.0089.

#### Methyl (2*S*,3*S*)-2-(acryloylamino)-3-methylpentanoate (6ba)

Acryloyl chloride (84.0 μL, 1.00 mmol) was dissolved in HCCl_3_ (13 mL) and cooled to 0 °C. A solution of l-isoleucine methyl ester hydrochloride (185 mg, 1.00 mmol) and NEt_3_ (295 μL, 2.10 mmol) in HCCl_3_ (13 mL) was added dropwise. After 1 h, mixture was concentrated under vacuum and the residue column chromatographed (silica gel, hexane/EtOAc 70 : 30) to give 6ba (154 mg, 0.770 mmol, 77%) as a pale yellow oil. [*α*]^26^_D_ + 30.8 (c 1.1, HCCl_3_). FTIR (ATR): *ν̃*_max_/cm^−1^ 3286 (NH), 2965, 2937, 2879, 1741 (C=O), 1657 (C=O), 1532 (NH). ^1^H NMR (500 MHz, CDCl_3_): *δ* (ppm) 6.31 (dd, *J* = 17.0, 1.3 Hz, 1H), 6.15 (dd, *J* = 17.0, 10.3 Hz, 1H), 6.11 (s, 1H, N–H), 5.68 (dd, *J* = 10.3, 1.0 Hz, 1H), 4.70 (dd, *J* = 8.6, 5.0 Hz, 1H), 3.75 (s, 3H, OCH_3_), 1.97–1.88 (m, 1H), 1.51–1.41 (m, 1H), 1.27–1.14 (m, 1H), 0.93 (t, *J* = 7.4 Hz, 3H), 0.91 (d, *J* = 6.9 Hz, 3H). ^13^C NMR (125 MHz, CDCl_3_) *δ* 172.65 (C=O), 165.21 (C=O), 130.55 (=CH), 127.35 (=CH_2_), 56.48 (CH), 52.32 (OMe), 38.31 (CH), 25.41 (CH_2_), 15.52 (CH_3_), 11.72 (CH_3_). HRMS (+ESI): *m*/*z* calcd for C_10_H_18_NO_3_^+^ [M + H]^+^: 200.1281; found: 200.1279.

#### Methyl (2*S*,3*S*)-3-methyl-2-[({3-oxo-2-[(2*Z*)-pent-2-en-1-yl]pyrazolidin-1-yl}acetyl)amino]pentanoate (3aaa)

Under an Ar atmosphere, 10a (100 mg, 0.632 mmol) and 6aa (258 mg, 0.822 mmol, 1.3 eq.) were dissolved in anhydrous DMF (1.0 mL). Cs_2_CO_3_ (340 mg, 0.960 mmol, 1.5 eq.) and 4 Å molecular sieves (580 mg) were added, and the resulting mixture was stirred at rt for 48 h. The reaction was left to settle, decanted, and the supernatant was concentrated under vacuum. The resulting crude oil was purified by column chromatography (silica gel, EtOAc) to give 3aaa (149 mg, 0.439 mmol, 70%) as an orange oil. [*α*]^23^_D_ + 13.4 (c 1.0, HCCl_3_). FTIR (ATR): *ν̃*_max_/cm^−1^ 3310 (NH), 3021 (C=CH), 2964, 2935, 2878, 1740 (C=O), 1683 (C=O), 1669 (C=O), 1513 (NH), 1204. ^1^H NMR (500 MHz, CDCl_3_): *δ* (ppm) 7.22 (d, *J* = 8.9 Hz, 1H, N–H), 5.67–5.59 (m, 1H), 5.43–5.35 (m, 1H), 4.61 (dd, *J* = 9.0, 4.8 Hz, 1H), 4.20–3.97 (m, 2H), 3.74 (s, 3H, OCH_3_), 3.53 (d, *J* = 15.8 Hz, 1H), 3.46 (d, *J* = 15.9 Hz, 1H), 3.48–3.20 (m, 2H), 2.74–2.47 (m, 2H), 2.14 (pd, *J* = 7.5, 0.8 Hz, 2H), 1.99–1.89 (m, 1H), 1.48–1.38 (m, 1H), 1.21–1.10 (m, 1H), 0.98 (t, *J* = 7.5 Hz, 3H), 0.93 (t, *J* = 7.4 Hz, 3H), 0.92 (d, *J* = 6.9 Hz, 3H). ^13^C NMR (125 MHz, CDCl_3_): *δ* (ppm) 172.19 (C=O), 171.23 (C=O), 168.23 (C=O), 136.44 (=CH), 122.75 (=CH), 58.66 (CH_2_), 56.14 (CH), 52.32 (OCH_3_), 51.47 (CH_2_), 39.61 (CH_2_), 37.79 (CH), 29.03 (CH_2_), 25.23 (CH_2_), 20.85 (CH_2_), 15.74 (CH_3_), 14.21 (CH_3_), 11.68 (CH_3_). HRMS (+ESI): *m*/*z* calcd for C_17_H_30_N_3_O_4_^+^ [M + H]^+^: 340.2231; found: 340.2224.

#### Methyl (2*S*)-4-methyl-2-[({3-oxo-2-[(2*Z*)-pent-2-en-1-yl]pyrazolidin-1-yl}acetyl)amino]pentanoate (3aab)

Compound 3aab was prepared from 10a (100 mg, 0.632 mmol) and 6ab (258 mg, 0.822 mmol), as described for 3aaa. Yield: 136 mg (0.401 mmol, 63%), orange oil. [*α*]^23^_D_ + 2.9 (c 1.0, HCCl_3_). FTIR (ATR): *ν̃*_max_/cm^−1^ 3303 (NH), 3019 (C=CH), 2958, 2935, 2873, 1744 (C=O), 1689 (C=O), 1667 (C=O), 1516 (NH), 1204. ^1^H NMR (500 MHz, CDCl_3_): *δ* (ppm) 7.05 (d, *J* = 8.9 Hz, 1H, N–H), 5.67–5.58 (m, 1H), 5.43–5.35 (m, 1H), 4.67 (td, *J* = 8.9, 5.0 Hz, 1H), 4.20–3.95 (m, 2H), 3.74 (s, 3H, OCH_3_), 3.52 (d, *J* = 15.8 Hz, 1H), 3.46 (d, *J* = 15.8 Hz, 1H), 3.47–3.20 (m, 2H), 2.75–2.46 (m, 2H), 2.12 (p, *J* = 7.5 Hz, 2H), 1.74–1.53 (m, 3H), 0.99 (t, *J* = 7.5 Hz, 3H), 0.95 (d, *J* = 6.3 Hz, 6H). ^13^C NMR (125 MHz, CDCl_3_): *δ* (ppm) 173.25 (C=O), 171.29 (C=O), 168.31 (C=O), 136.46 (=CH), 122.82 (=CH), 58.72 (CH_2_), 52.53 (OCH_3_), 51.51 (CH_2_), 50.38 (CH), 41.46 (CH_2_), 39.62 (CH_2_), 29.06 (CH_2_), 25.17 (CH), 23.00 (CH_3_), 21.94 (CH_3_), 20.88 (CH_2_), 14.24 (CH_3_). HRMS (+ESI): *m*/*z* calcd for C_17_H_30_N_3_O_4_^+^ [M + H]^+^: 340.2231; found: 340.2226.

#### Methyl (2*S*)-3-methyl-2-[({3-oxo-2-[(2*Z*)-pent-2-en-1-yl]pyrazolidin-1-yl}acetyl)amino]butanoate (3aac)

Compound 3aac was prepared from 10a (68.0 mg, 0.429 mmol) and 6ac (167 mg, 0.558 mmol), as described for 3aaa. Yield 107 mg (0.329 mmol, 77%), orange oil. [*α*]^24^_D_ + 9.6 (c 1.1, HCCl_3_). FTIR (ATR): *ν̃*_max_/cm^−1^ 3308 (NH), 3020 (C=CH), 2964, 2935, 2876, 1741 (C=O), 1683 (C=O), 1667 (C=O), 1513 (NH), 1206. ^1^H NMR (500 MHz, CDCl_3_): *δ* (ppm) 7.21 (d, *J* = 9.0 Hz, 1H, N–H), 5.67–5.60 (m, 1H), 5.43–5.36 (m, 1H), 4.57 (dd, *J* = 9.2, 4.7 Hz, 1H), 4.21–3.97 (m, 2H), 3.74 (s, 3H, OCH_3_), 3.54 (d, *J* = 15.8 Hz, 1H), 3.46 (d, *J* = 15.9 Hz, 1H), 3.47–3.20 (m, 2H), 2.74–2.47 (m, 2H), 2.22 (septd, *J* = 6.9, 4.9 Hz, 1H), 2.12 (pd, *J* = 7.5, 1.0 Hz, 2H), 0.98 (t, *J* = 7.5 Hz, 3H), 0.95 (d, *J* = 6.9 Hz, 3H), 0.90 (d, *J* = 6.9 Hz, 3H). ^13^C NMR (125 MHz, CDCl_3_): *δ* (ppm) 172.21 (C=O), 171.24 (C=O), 168.37 (C=O), 136.48 (=CH), 122.75 (=CH), 58.66 (CH_2_), 56.72 (CH), 52.39 (OCH_3_), 51.49 (CH_2_), 39.61 (CH_2_), 31.14 (CH), 29.04 (CH_2_), 20.87 (CH_2_), 19.23 (CH_3_), 17.78 (CH_3_), 14.24 (CH_3_). HRMS (+ESI): *m*/*z* calcd for C_16_H_28_N_3_O_4_^+^ [M + H]^+^: 326.2074; found: 326.2069.

#### Methyl (2*S*)-2-[({3-oxo-2-[(2*Z*)-pent-2-en-1-yl]pyrazolidin-1-yl}acetyl)amino]propanoate (3aad)

Compound 3aad was prepared from 10a (100 mg, 0.632 mmol) and 6ad (223 mg, 0.822 mmol), as described for 3aaa. Yield: 89.8 mg (0.303 mmol, 48%), orange oil. [*α*]^24^_D_ + 5.3 (c 1.0, HCCl_3_). FTIR (ATR): *ν̃*_max_/cm^−1^ 3307 (NH), 3062 (C=CH), 2956, 2941, 2876, 1743 (C=O), 1683 (C=O), 1663 (C=O), 1520 (NH), 1209. ^1^H NMR (300 MHz, CDCl_3_): *δ* (ppm) 7.20 (d, *J* = 7.7 Hz, 1H, N–H), 5.58–5.48 (m, 1H), 5.34–5.22 (m, 1H), 4.59–4.46 (m, 1H), 4.08–3.87 (m, 2H), 3.66 (s, 3H, OCH_3_), 3.44 (d, *J* = 15.9 Hz, 1H), 3.37 (d, *J* = 16.0 Hz, 1H), 3.35–3.14 (m, 2H), 2.64–2.38 (m, 2H), 2.02 (p, *J* = 7.3 Hz, 2H), 1.33 (d, *J* = 7.2 Hz, 3H), 0.89 (t, *J* = 7.5 Hz, 3H). ^13^C NMR (75 MHz, CDCl_3_): *δ* (ppm) 172.84 (C=O), 171.17 (C=O), 167.93 (C=O), 136.12 (=CH), 122.53 (=CH), 58.62 (CH_2_), 52.39 (OCH_3_), 51.36 (CH_2_), 47.47 (CH), 39.36 (CH_2_), 28.87 (CH_2_), 20.57 (CH_2_), 18.03 (CH_3_), 13.92 (CH_3_). HRMS (+ESI): *m*/*z* calcd for C_14_H_23_N_3_O_4_^+^ [M + H]^+^: 298.1761; found: 298.1758.

#### (2*S*,3*S*)-3-Methyl-2-[({3-oxo-2-[(2*Z*)-pent-2-en-1-yl]pyrazolidin-1-yl}acetyl)amino]pentanoic acid (3aae)

Under an Ar atmosphere, 10a (36.0 mg, 0.222 mmol) and 6ae (86.0 mg, 0.285 mmol) were dissolved in anhydrous DMF (1.0 mL) and stirred at rt for 16 h. Cs_2_CO_3_ (40.0 mg, 0.113 mmol) was added and stirring was continued for 1 h. DMF was removed under high vacuum, and the residue was redispersed a couple of times in DCM (1.0 mL) and concentrated again under reduced pressure. The resulting crude oil was purified by column chromatography (silica gel, DCM/MeOH 95.2 : 4.8) to give the acid 3aae (42.0 mg, 0.129 mmol, 58%) as an orange oil. [*α*]^23^_D_ + 1.0 (c 0.7, HCCl_3_). FTIR (ATR): *ν̃*_max_/cm^−1^ 3300–2700 (COOH), 3290 (NH), 3061 (C=CH), 2964, 2934, 2877, 1730 (C=O), 1653 (C=O), 1526 (NH). ^1^H NMR (300 MHz, CDCl_3_): *δ* (ppm) 7.26 (d, *J* = 8.4 Hz, 1H, N–H), 5.68–5.58 (m, 1H), 5.43–5.33 (m, 1H), 4.65–4.49 (m, 1H), 4.21–3.94 (m, 2H), 3.56 (d, *J* = 15.8 Hz, 1H), 3.49 (d, *J* = 16.0 Hz, 1H), 3.47–3.24 (m, 2H), 2.78–2.50 (m, 2H), 2.11 (p, *J* = 7.5 Hz, 2H), 2.05–1.91 (m, 2H), 1.56–1.43 (m, 1H), 1.32–1.13 (m, 1H), 1.01–0.88 (m, 9H). ^13^C NMR (125 MHz, CDCl_3_): *δ* (ppm) 174.49 (C=O), 171.72 (C=O), 168.81 (C=O), 136.57 (=CH), 122.55 (=CH), 58.44 (CH_2_), 56.44 (CH), 51.47 (CH_2_), 39.69 (CH_2_), 37.44 (CH), 29.13 (CH_2_), 25.12 (CH_2_), 20.86 (CH_2_), 15.79 (CH_3_), 14.20 (CH_3_), 11.74 (CH_3_). HRMS (+ESI): *m*/*z* calcd for C_16_H_28_N_3_O_4_^+^ [M + H]^+^: 326.2074; found: 326.2070.

#### (2*S*,3*S*)-3-Methyl-2-{[(3-oxo-2-pentylpyrazolidin-1-yl)acetyl]amino}pentanoate (3baa)

Compound 3baa was prepared from 10b (100 mg, 0.640 mmol) and 6aa (300 mg, 0.960 mmol), as described for 3aaa. Yield 181 mg (0.530 mmol, 83%), orange oil. [*α*]^24^_D_ + 12.7 (c 1.0, HCCl_3_). FTIR (ATR): *ν̃*_max_/cm^−1^ 3308 (NH), 2960, 2934, 2875, 1741 (C=O), 1681 (C=O), 1665 (C=O), 1513 (NH), 1204. ^1^H NMR (500 MHz, CDCl_3_): *δ* (ppm) 7.27 (d, *J* = 8.9 Hz, 1H, N–H), 4.63 (dd, *J* = 9.0, 4.7 Hz, 1H), 3.75 (s, 3H, OCH_3_), 3.48 (d, *J* = 16.1 Hz, 1H), 3.43 (d, *J* = 16.1 Hz, 1H), 3.43–3.25 (m, 4H), 2.71–2.51 (m, 2H), 2.01–1.91 (m, 1H), 1.68–1.58 (m, 2H), 1.50–1.39 (m, 1H), 1.39–1.23 (m, 4H), 1.23–1.11 (m, 1H), 0.94 (t, *J* = 7.4 Hz, 3H), 0.93 (d, *J* = 6.9 Hz, 3H), 0.90 (t, *J* = 7.1 Hz, 3H). ^13^C NMR (125 MHz, CDCl_3_): *δ* (ppm) 172.23 (C=O), 171.22 (C=O), 168.28 (C=O), 58.35 (CH_2_), 56.15 (CH), 52.37 (OCH_3_), 51.56 (CH_2_), 41.94 (CH_2_), 37.83 (CH), 29.30 (CH_2_), 28.98 (CH_2_), 27.12 (CH_2_), 25.27 (CH_2_), 22.45 (CH_2_), 15.80 (CH_3_), 14.09 (CH_3_), 11.71 (CH_3_). HRMS (+ESI): *m*/*z* calcd for C_17_H_32_N_3_O_4_^+^ [M + H]^+^: 342.2387; found: 342.2381.

#### (2*S*,3*S*)-2-{[(2-Hexyl-3-oxopyrazolidin-1-yl)acetyl]amino}-3-methylpentanoate (3caa)

Compound 3caa was prepared from 10c (87.0 mg, 0.511 mmol) and 6aa (208 mg, 0.664 mmol), as described for 3aaa. Yield: 120 mg (0.338 mmol, 66%), yellow oil. [*α*]^25^_D_ + 11.9 (c 1.0, HCCl_3_). FTIR (ATR): *ν̃*_max_/cm^−1^ 3308 (NH), 2959, 2933, 2858, 1741 (C=O), 1681 (C=O), 1665 (C=O), 1513 (NH), 1205. ^1^H NMR (500 MHz, CDCl_3_): *δ* (ppm) 7.27 (d, *J* = 9.3 Hz, 1H, N–H), 4.63 (dd, *J* = 9.0, 4.7 Hz, 1H), 3.75 (s, 3H, OCH_3_), 3.48 (d, *J* = 16.1 Hz, 1H), 3.43 (d, *J* = 16.1 Hz, 1H), 3.44–3.23 (m, 4H), 2.71–2.52 (m, 2H), 2.01–1.92 (m, 1H), 1.66–1.57 (m, 2H), 1.49–1.39 (m, 1H), 1.36–1.24 (m, 6H), 1.22–1.11 (m, 1H), 0.94 (t, *J* = 7.4 Hz, 3H), 0.94 (d, *J* = 6.9 Hz, 3H), 0.88 (t, *J* = 6.8 Hz, 3H). ^13^C NMR (125 MHz, CDCl_3_): *δ* (ppm) 172.24 (C=O), 171.22 (C=O), 168.29 (C=O), 58.36 (CH_2_), 56.15 (CH), 52.37 (OCH_3_), 51.57 (CH_2_), 41.99 (CH_2_), 37.82 (CH), 31.57 (CH_2_), 29.31 (CH_2_), 27.40 (CH_2_), 26.51 (CH_2_), 25.27 (CH_2_), 22.64 (CH_2_), 15.81 (CH_3_), 14.14 (CH_3_), 11.73 (CH_3_). HRMS (+ESI): *m*/*z* calcd for C_18_H_34_N_3_O_4_^+^ [M + H]^+^: 356.2544; found: 356.2534.

#### Methyl (2*S*,3*S*)-2-{[(2-butyl-3-oxopyrazolidin-1-yl)acetyl]amino}-3-methylpentanoate (3daa)

Compound 3daa was prepared from 10d (70.0 mg, 0.492 mmol) and 6aa (200 mg, 0.640 mmol), as described for 3aaa. Yield: 119 mg (0.363 mmol, 74%), orange oil. [*α*]^25^_D_ + 12.6 (c 1.0, HCCl_3_). FTIR (ATR): *ν̃*_max_/cm^−1^ 3304 (NH), 2961, 2935, 2876, 1741 (C=O), 1683 (C=O), 1663 (C=O), 1513 (NH), 1204. ^1^H NMR (500 MHz, CDCl_3_): *δ* (ppm) 7.28 (d, *J* = 8.9 Hz, 1H, N–H), 4.63 (dd, *J* = 9.0, 4.7 Hz, 1H), 3.75 (s, 3H, OCH_3_), 3.49 (d, *J* = 16.1 Hz, 1H), 3.43 (d, *J* = 16.1 Hz, 1H), 3.45–3.23 (m, 4H), 2.71–2.49 (m, 2H), 2.01–1.91 (m, 1H), 1.65–1.57 (m, 2H), 1.50–1.40 (m, 1H), 1.35 (sext, *J* = 7.5 Hz, 2H), 1.22–1.12 (m, 1H), 0.94 (t, *J* = 6.8 Hz, 6H), 0.93 (d, *J* = 6.8 Hz, 3H). ^13^C NMR (125 MHz, CDCl_3_): *δ* (ppm) 172.25 (C=O), 171.23 (C=O), 168.27 (C=O), 58.36 (CH_2_), 56.15 (CH), 52.38 (OCH_3_), 51.57 (CH_2_), 41.67 (CH_2_), 37.85 (CH), 29.53 (CH_2_), 29.30 (CH_2_), 25.29 (CH_2_), 20.08 (CH_2_), 15.80 (CH_3_), 13.87 (CH_3_), 11.73 (CH_3_). HRMS (+ESI): *m*/*z* calcd for C_16_H_30_N_3_O_4_^+^ [M + H]^+^: 328.2231; found: 328.2224.

#### Methyl (2*S*,3*S*)-2-{[3-(2-butyl-3-oxopyrazolidin-1-yl)propanoyl]amino}-3-methylpentanoate (3aba)

10a (31.0 mg, 0.197 mmol) and 6ba (60.0 mg, 0.296 mmol) were dissolved in a 1 : 1H_2_O/trifluoroethanol (TFE) biphasic system (1.0 mL). This mixture was heated at 100 °C for 24 h. At rt, acetone was added (5 mL) and the resulting solution was concentrated under reduced pressure to give an orange oil. The Michael adduct was purified by column chromatography (silica gel, DCM/MeOH/NH_3_ (aq., 25%) 97.1 : 2.6 : 0.3) providing 3aba (40.0 mg, 0.113 mmol, 58%) as a brown oil. [*α*]^25^_D_ + 15.5 (c 1.0, HCCl_3_). FTIR (ATR): *ν̃*_max_/cm^−1^ 3293 (NH), 3021 (C=CH), 2964, 2935, 2878, 1742 (C=O), 1667 (C=O), 1652 (C=O), 1535 (NH), 1201. ^1^H NMR (500 MHz, CDCl_3_): *δ* (ppm) 6.77 (d, *J* = 8.0 Hz, 1H, H–N), 5.60–5.52 (m, 1H), 5.43–5.36 (m, 1H), 4.59 (dd, *J* = 8.5, 5.0 Hz, 1H), 4.26–3.95 (m, 2H), 3.73 (s, 3H, OCH_3_), 3.40–3.00 (m, 4H), 2.71–2.45 (m, 2H), 2.44 (t, *J* = 6.8 Hz, 2H), 2.13 (pd, *J* = 7.5, 0.8 Hz, 2H), 1.93–1.83 (m, 1H), 1.48–1.38 (m, 1H), 1.22–1.10 (m, 1H), 0.98 (t, *J* = 7.5 Hz, 3H), 0.92 (t, *J* = 7.3 Hz, 3H), 0.90 (d, *J* = 6.8 Hz, 3H). ^13^C NMR (125 MHz, CDCl_3_): *δ* (ppm) 172.61 (C=O), 171.50 (C=O), 170.61 (C=O), 135.68 (CH), 123.32 (CH), 56.54 (CH), 52.26 (OCH_3_), 51.39 (CH_2_), 49.16 (CH_2_), 40.08 (CH_2_), 37.99 (CH), 33.84 (CH_2_), 29.66 (CH_2_), 25.45 (CH_2_), 20.96 (CH_2_), 15.61 (CH_3_), 14.20 (CH_3_), 11.73 (CH_3_). HRMS (+ESI): *m*/*z* calcd for C_18_H_32_N_3_O_4_^+^ [M + H]^+^: 354.2387; found: 354.2378.

#### Methyl (2*S*)-4-methyl-2-{[(3-oxo-2-pentylpyrazolidin-1-yl)acetyl]amino}pentanoate (3bab)

Compound 3bab was prepared from 10b (15.8 mg, 0.101 mmol) and 6ac (47.0 mg, 0.150 mmol), as described for 3aaa. Yield 17.9 mg (0.0524 mmol, 52%), orange oil. FTIR (ATR): *ν̃*_max_/cm^−1^ 3297 (NH), 2955, 2933, 2869, 1743 (C=O), 1661 (C=O), 1519 (NH), 1204. ^1^H NMR (500 MHz, CDCl_3_): *δ* 7.09 (d, *J* = 8.6 Hz, 1H), 4.71–4.64 (m, 1H), 3.74 (s, 3H), 3.47 (d, *J* = 16.0 Hz, 1H), 3.43 (d, *J* = 16.1 Hz, 1H), 3.47–3.20 (m, 4H), 2.72–2.47 (m, 2H), 1.80–1.53 (m, 5H), 1.39–1.22 (m, 4H), 0.95 (d, *J* = 6.3 Hz, 6H), 0.90 (t, *J* = 7.1 Hz, 3H). ^13^C NMR (125 MHz, CDCl_3_) *δ* 173.26 (C=O), 171.27 (C=O), 168.33 (C=O), 58.41 (CH_2_), 52.54 (OCH_3_), 51.55 (CH_2_), 50.42 (CH), 42.00 (CH_2_), 41.47 (CH_2_), 29.29 (CH_2_), 28.97 (CH_2_), 27.16 (CH_2_), 25.21 (CH), 22.96 (CH_3_), 22.43 (CH_2_), 22.01 (CH_3_), 14.09 (CH_3_). HRMS (+ESI): *m*/*z* calcd for C_17_H_32_N_3_O_4_^+^ [M + H]^+^: 342.2387; found: 342.2388.

#### Methyl (2*S*)-3-methyl-2-{[(3-oxo-2-pentylpyrazolidin-1-yl)acetyl]amino}butanoate (3bac)

Compound 3bac was prepared from 10b (75.3 mg, 0.482 mmol) and 6ac (144 mg, 0.482 mmol), as described for 3aaa. Yield 105 mg (0.321 mmol, 67%), orange oil. FTIR (ATR): *ν̃*_max_/cm^−1^ 3305 (NH), 2958, 2933, 2874, 1741 (C=O), 1684 (C=O), 1664 (C=O), 1515 (NH), 1208. ^1^H NMR (300 MHz, CDCl_3_): *δ* (ppm) 7.24 (d, *J* = 9.1 Hz, 1H), 4.53 (dd, *J* = 9.1, 4.7 Hz, 1H), 3.70 (s, 3H), 3.46 (d, *J* = 16.1 Hz, 1H), 3.39 (d, *J* = 16.2 Hz, 1H), 3.41–3.22 (m, 2H), 2.65–2.47 (m, 2H), 2.27–2.10 (m, 1H), 1.58 (p, *J* = 7.0 Hz, 2H), 1.35–1.18 (m, 4H), 0.92 (d, *J* = 6.9 Hz, 3H), 0.87 (d, *J* = 7.0 Hz, 3H), 0.85 (t, *J* = 6.9 Hz, 3H). ^13^C NMR (75 MHz, CDCl_3_) *δ* 172.10 (C=O), 171.15 (C=O), 168.33 (C=O), 58.20 (CH_2_), 56.61 (CH), 52.28 (OCH_3_), 51.43 (CH_2_), 41.82 (CH_2_), 31.03 (CH), 29.19 (CH_2_), 28.86 (CH_2_), 26.97 (CH_2_), 22.31 (CH_2_), 19.14 (CH_3_), 17.65 (CH_3_), 13.97 (CH_3_). HRMS (+ESI): *m*/*z* calcd for C_16_H_30_N_3_O_4_^+^ [M + H]^+^: 328.2231; found: 328.2237.

## Author contributions

Concept: W. Q. F. and D. D.; experiments: S. V. P, D. D., and C. J. M.; supervision and resources: M. B. and W. Q. F.; writing: S. V. P, M. B. and W. Q. F.

## Conflicts of interest

There are no conflicts to declare.

## Supplementary Material

RA-014-D3RA07887F-s001
