# Supplementary material for: Facile modular synthesis of jasmonoyl-l-isoleucine analogs possessing a pyrazolidin-3-one core
Source: RSC Adv. 2024 Jan 25;14(6):3790–7. doi: 10.1039/d3ra07887f (PMC10808958; doi:10.1039/d3ra07887f)

Electronic Supplementary Material (ESI) for RSC Advances.
This journal is © The Royal Society of Chemistry 2024

Facile Modular Synthesis of Jasmonoyl-L-Isoleucine Analogs Possessing a Pyrazolidin-3-one Core

Samuel Vizcaíno Páez,[a] Diego Durango Restrepo,[b] Christian Jürgen Müller,[c] Matthias Breuning[c*] and Wiston Quiñones Fletcher[a*]

[a] Química orgánica de productos naturales, Universidad de Antioquia, Medellín, 050010, Antioquia, Colombia

[b] Química de productos naturales y los alimentos, Universidad Nacional de Colombia, Medellín, 050034, Antioquia, Colombia

[c] Department of Chemistry, University of Bayreuth, Universitätsstraße 30, 95447 Bayreuth, Germany

NMR spectra

The spectra are given in the same order as in the experiments described in the main text. All signal assignments in the ^{1}H and ^{13}C NMR data were made on basis of 2D NMR spectra (COSY, HSQC, HMBC).

^{1}H NMR (500 MHz, $CDCl_3$) and ^{13}C Jmod NMR (125 MHz, $CDCl_3$) of **4**:

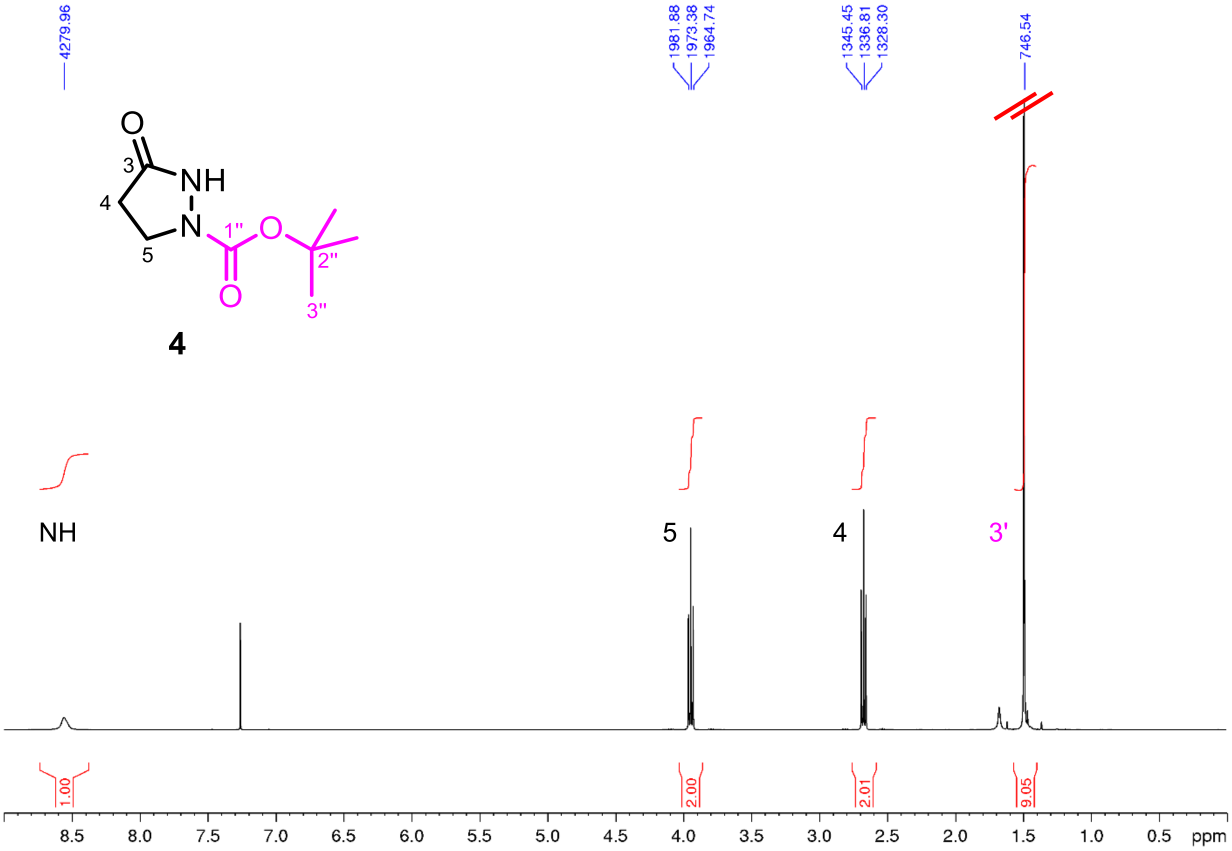

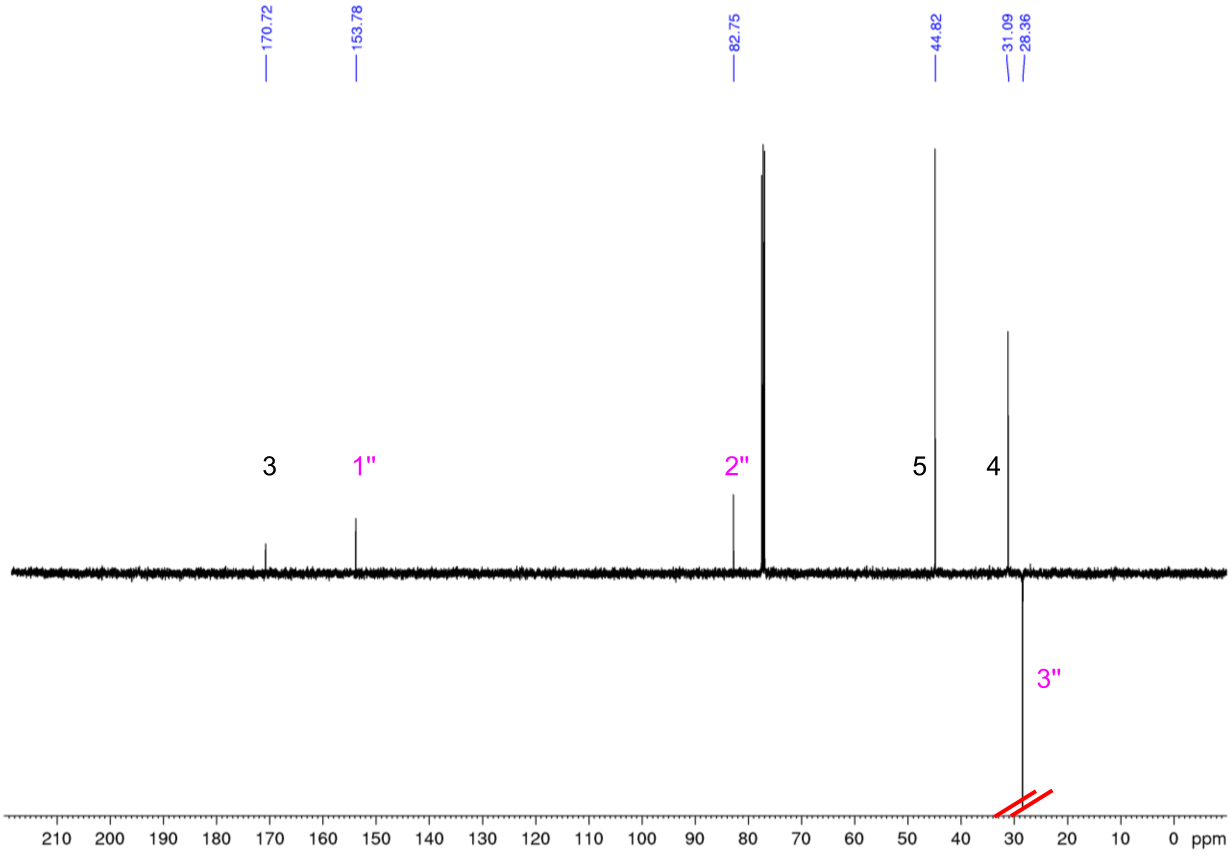

^{1}H NMR (500 MHz, $CDCl_3$) and ^{13}C Jmod NMR (125 MHz, $CDCl_3$) of **9a**:

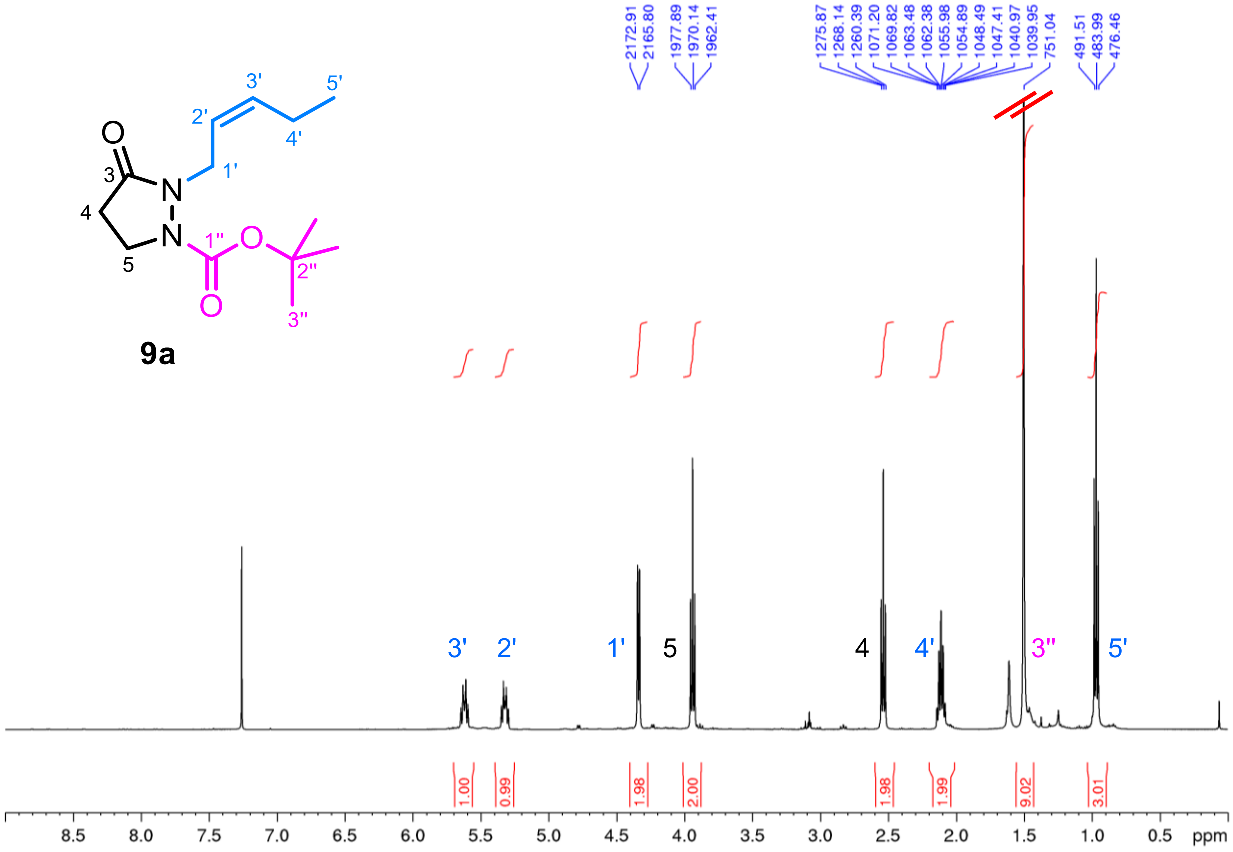

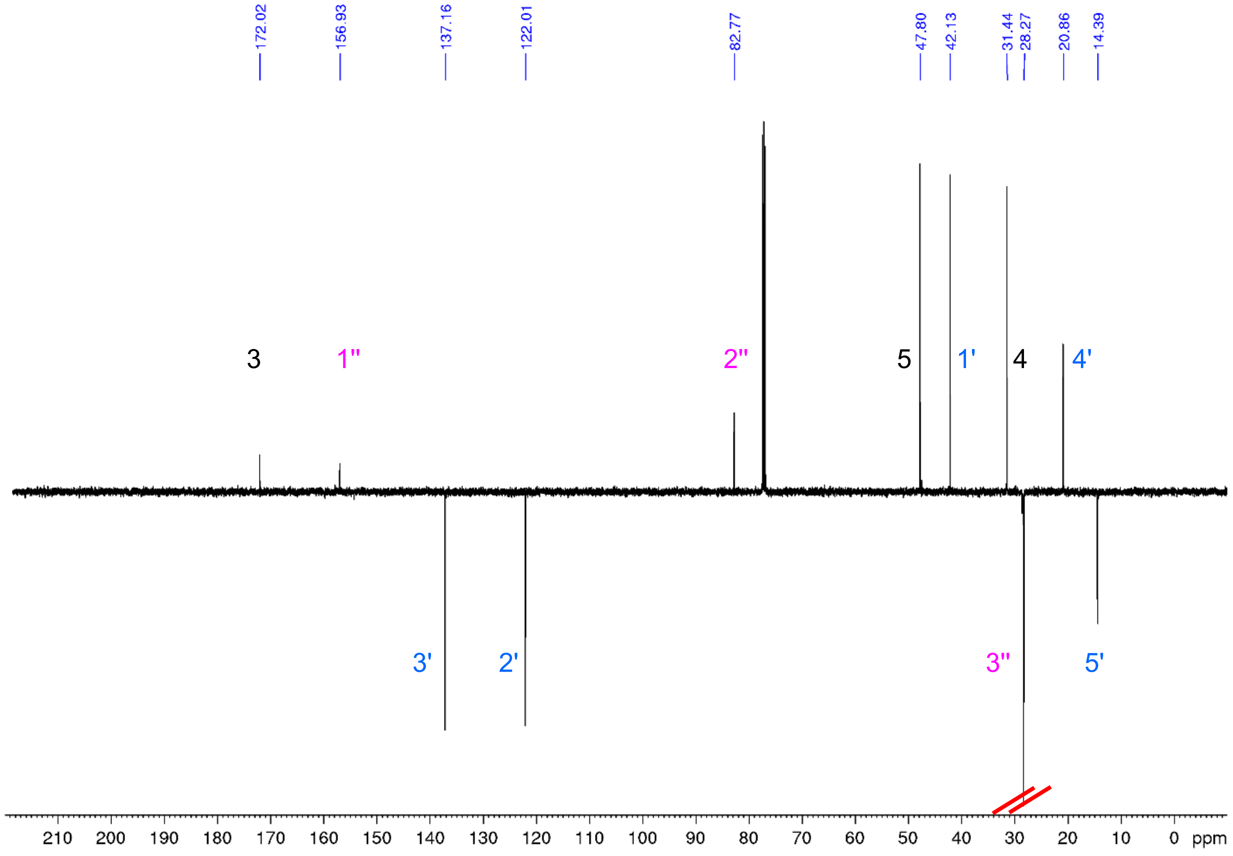

^1H NMR (500 MHz, $CDCl_3$) and ^{13}C Jmod NMR (125 MHz, $CDCl_3$) of **9b**:

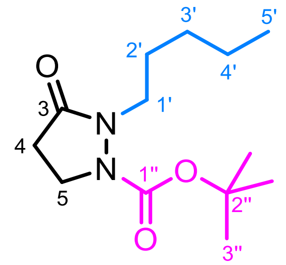

9b

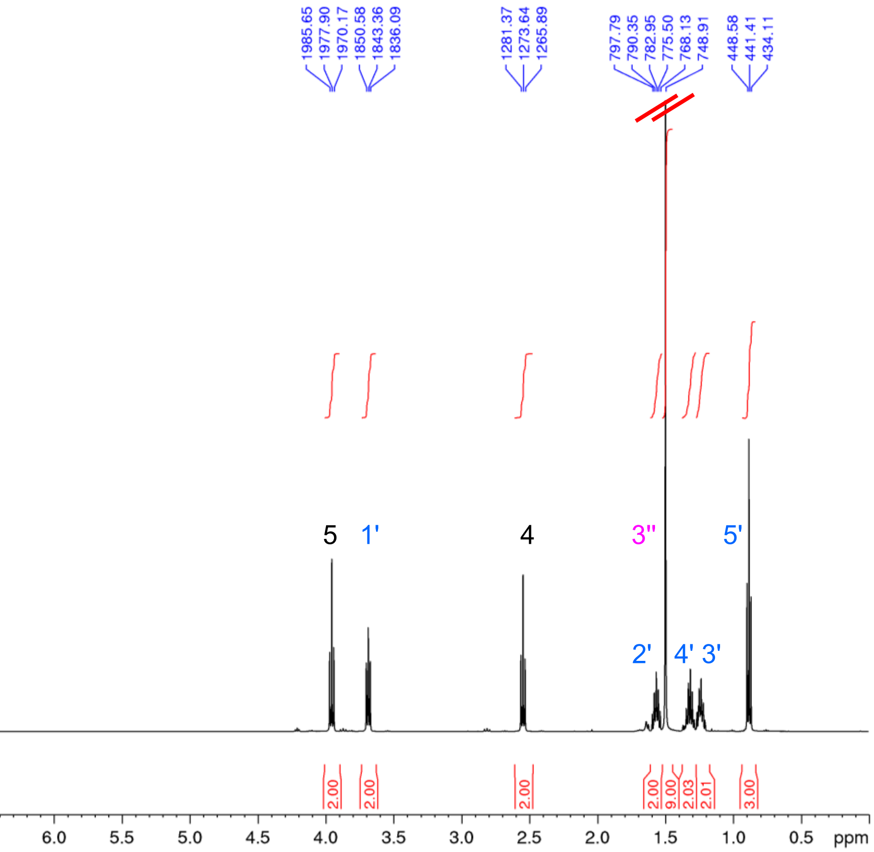

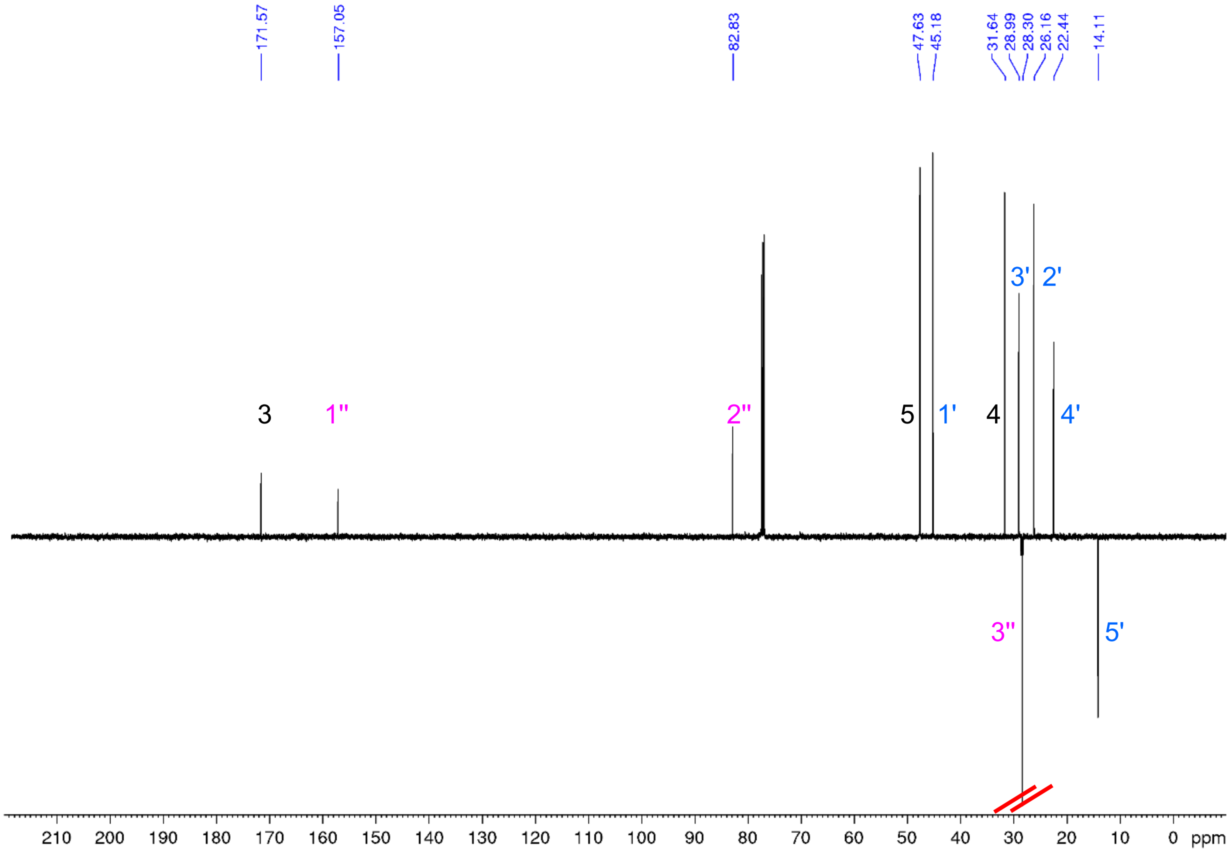

^{1}H NMR (500 MHz, $CDCl_3$) and ^{13}C Jmod NMR (125 MHz, $CDCl_3$) of **9c**:

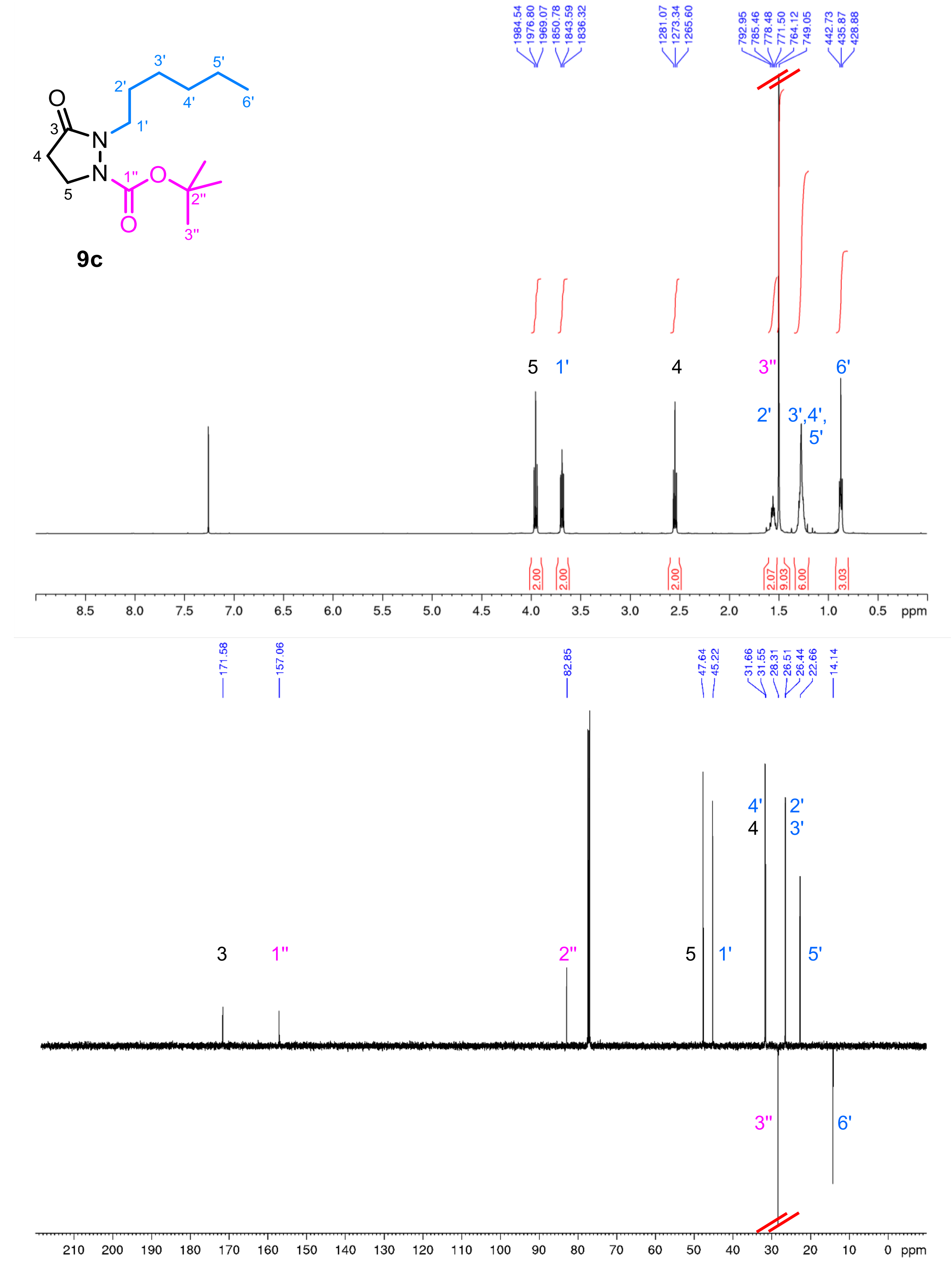

^{1}H NMR (500 MHz, $CDCl_3$) and ^{13}C Jmod NMR (125 MHz, $CDCl_3$) of **9d**:

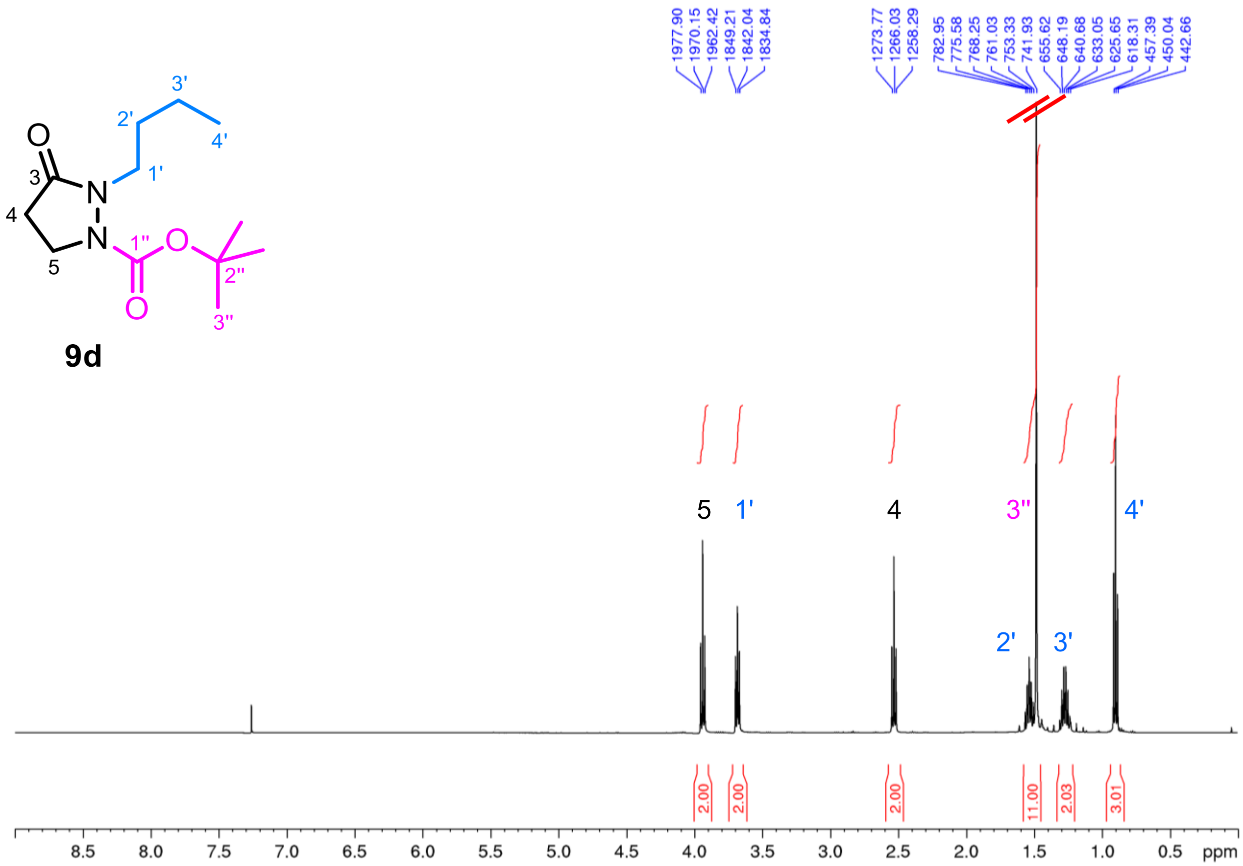

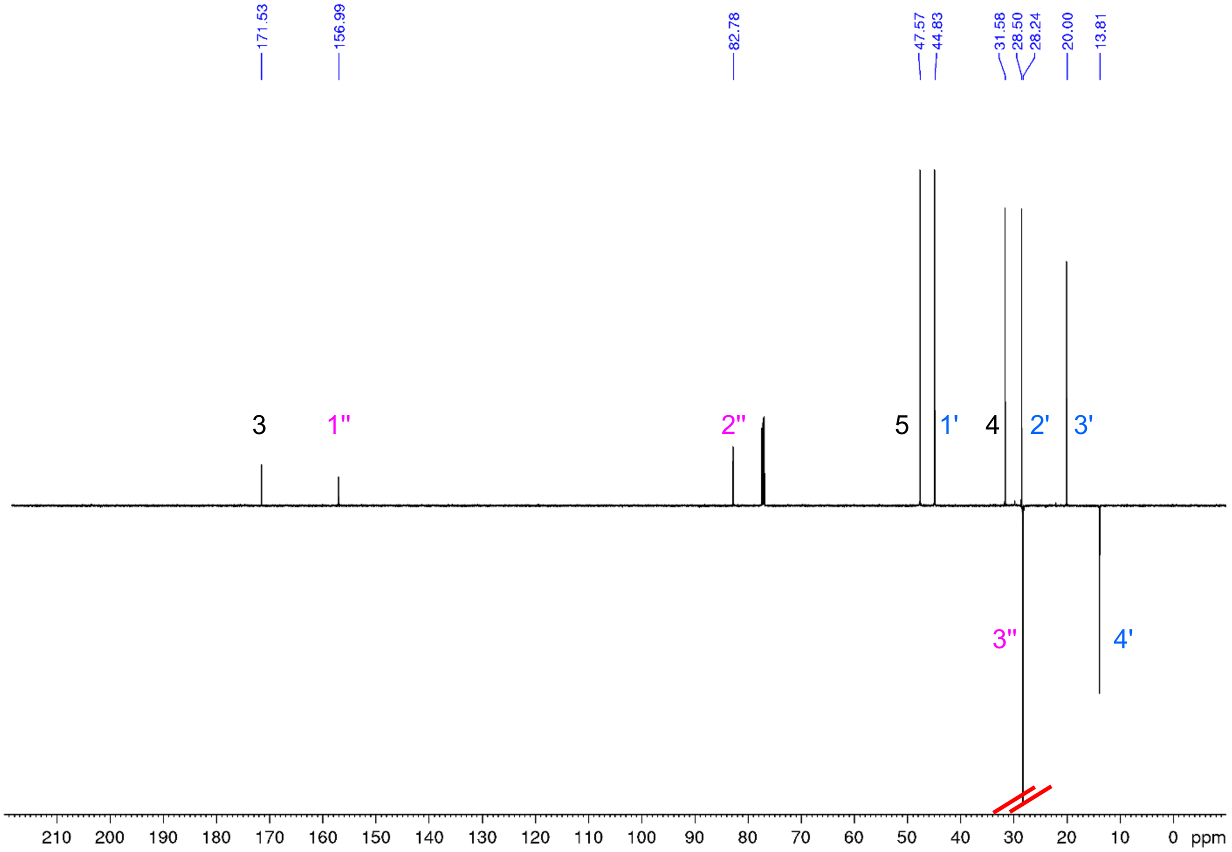

^{1}H NMR (500 MHz, $CDCl_3$) and ^{13}C Jmod NMR (125 MHz, $CDCl_3$) of **10a**:

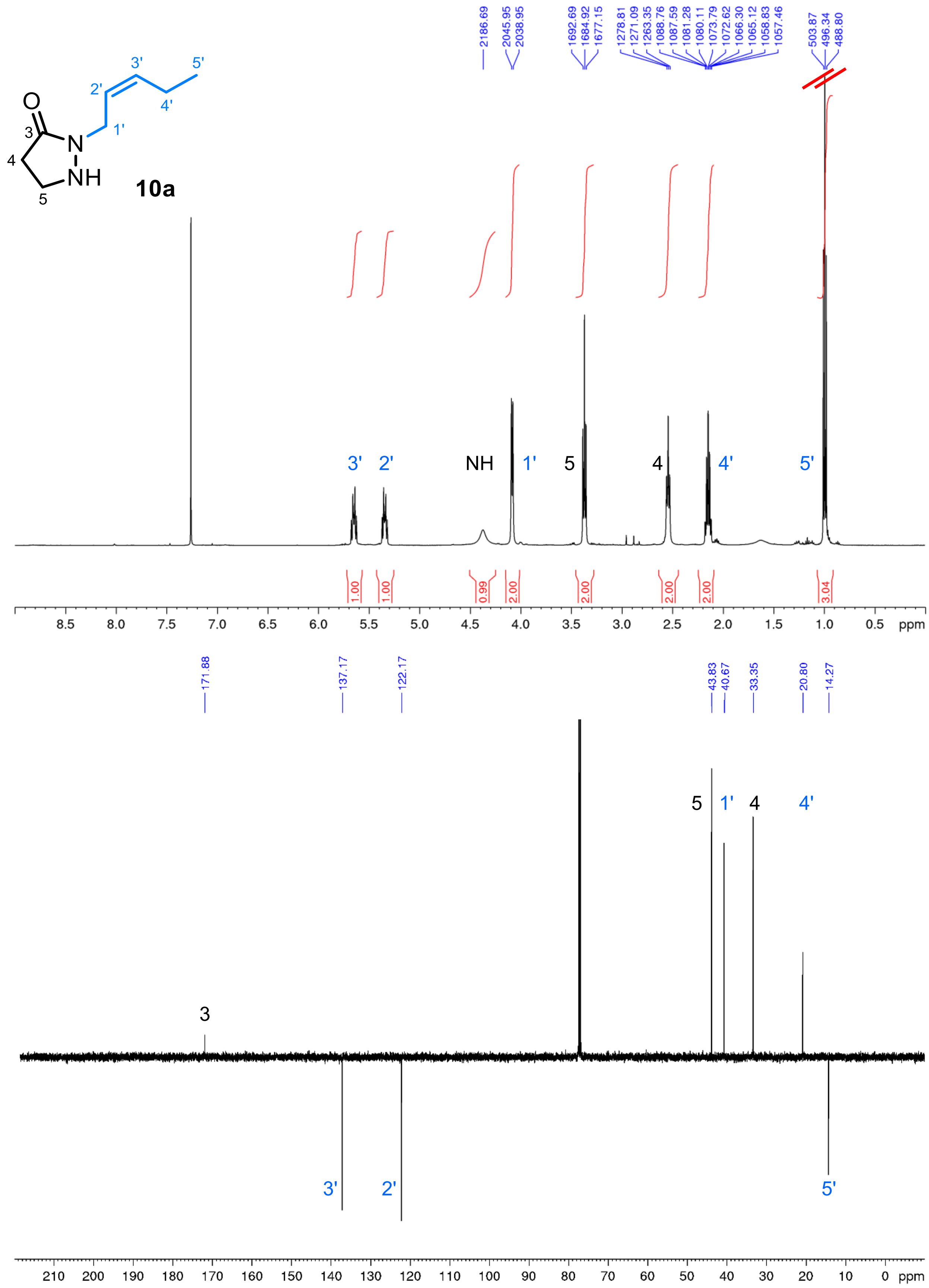

^{1}H NMR (500 MHz, $CDCl_3$) and ^{13}C Jmod NMR (125 MHz, $CDCl_3$) of **10b**:

10b

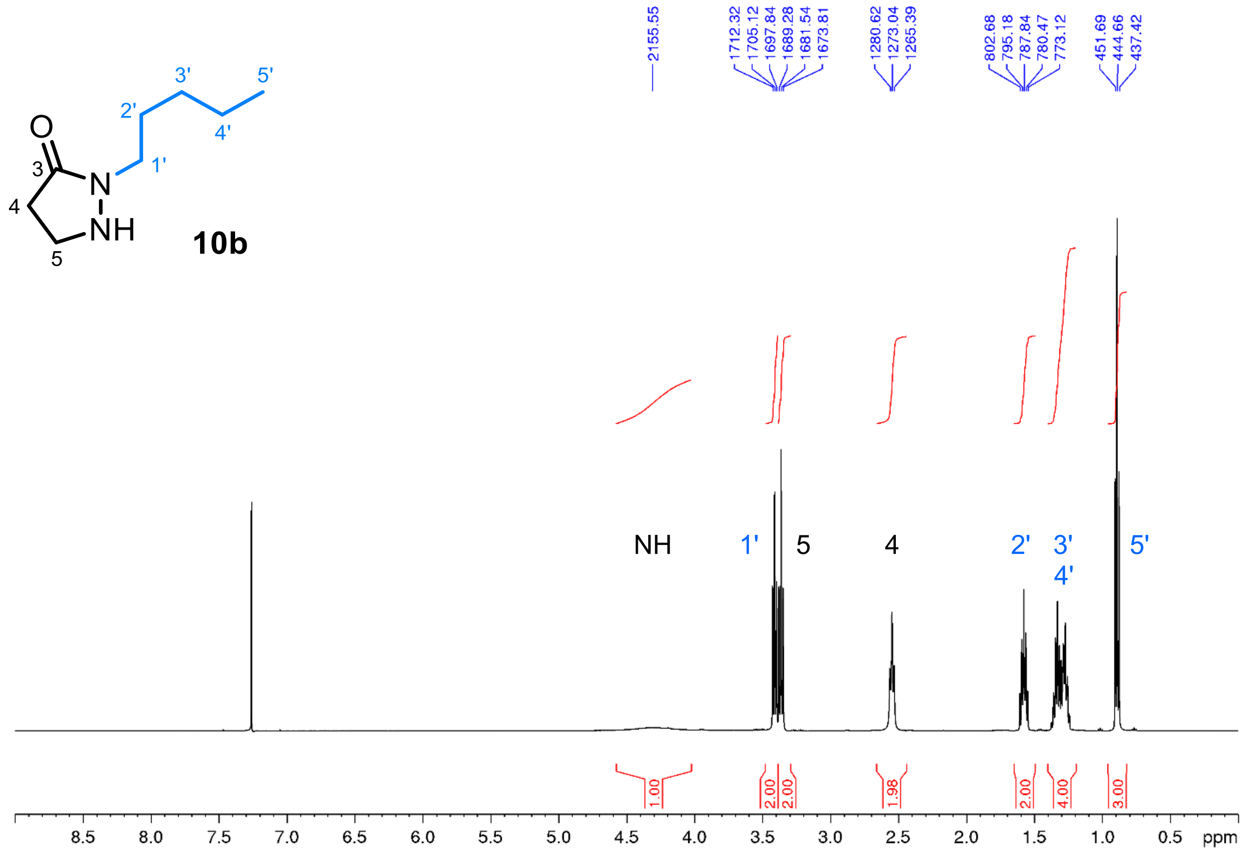

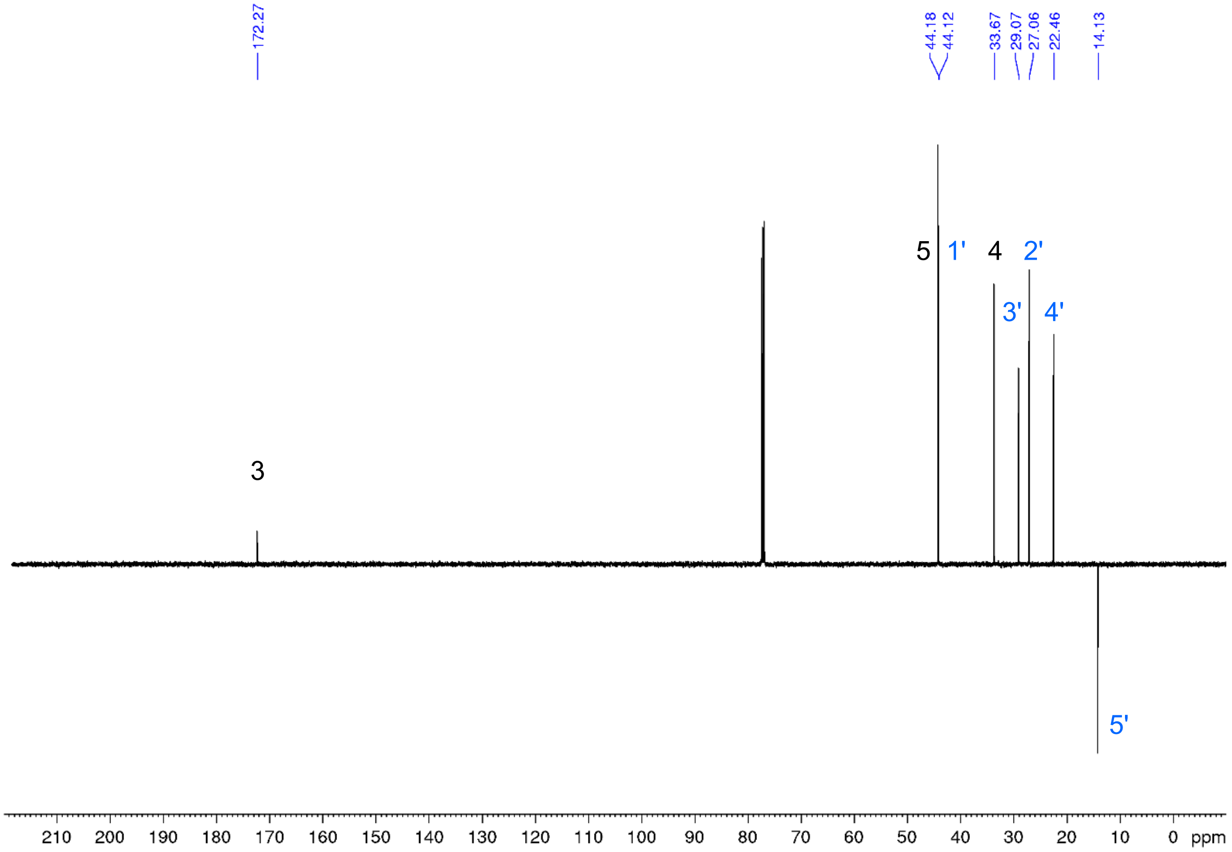

^{1}H NMR (500 MHz, $CDCl_3$) and ^{13}C Jmod NMR (125 MHz, $CDCl_3$) of **10c**:

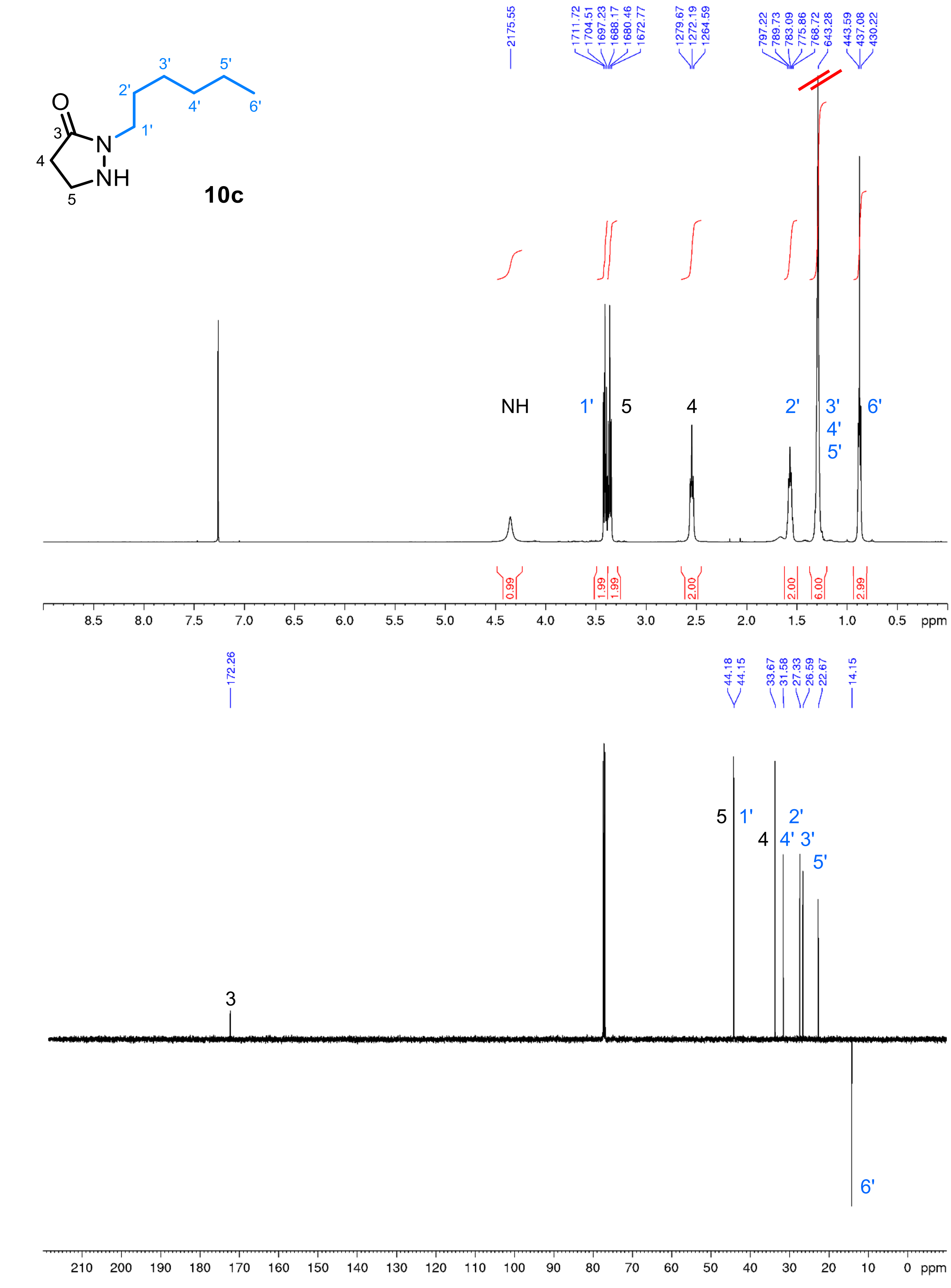

^{1}H NMR (500 MHz, $CDCl_3$) and ^{13}C Jmod NMR (125 MHz, $CDCl_3$) of **10d**:

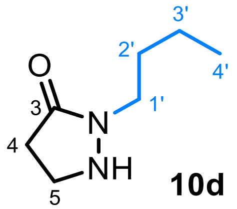

10d

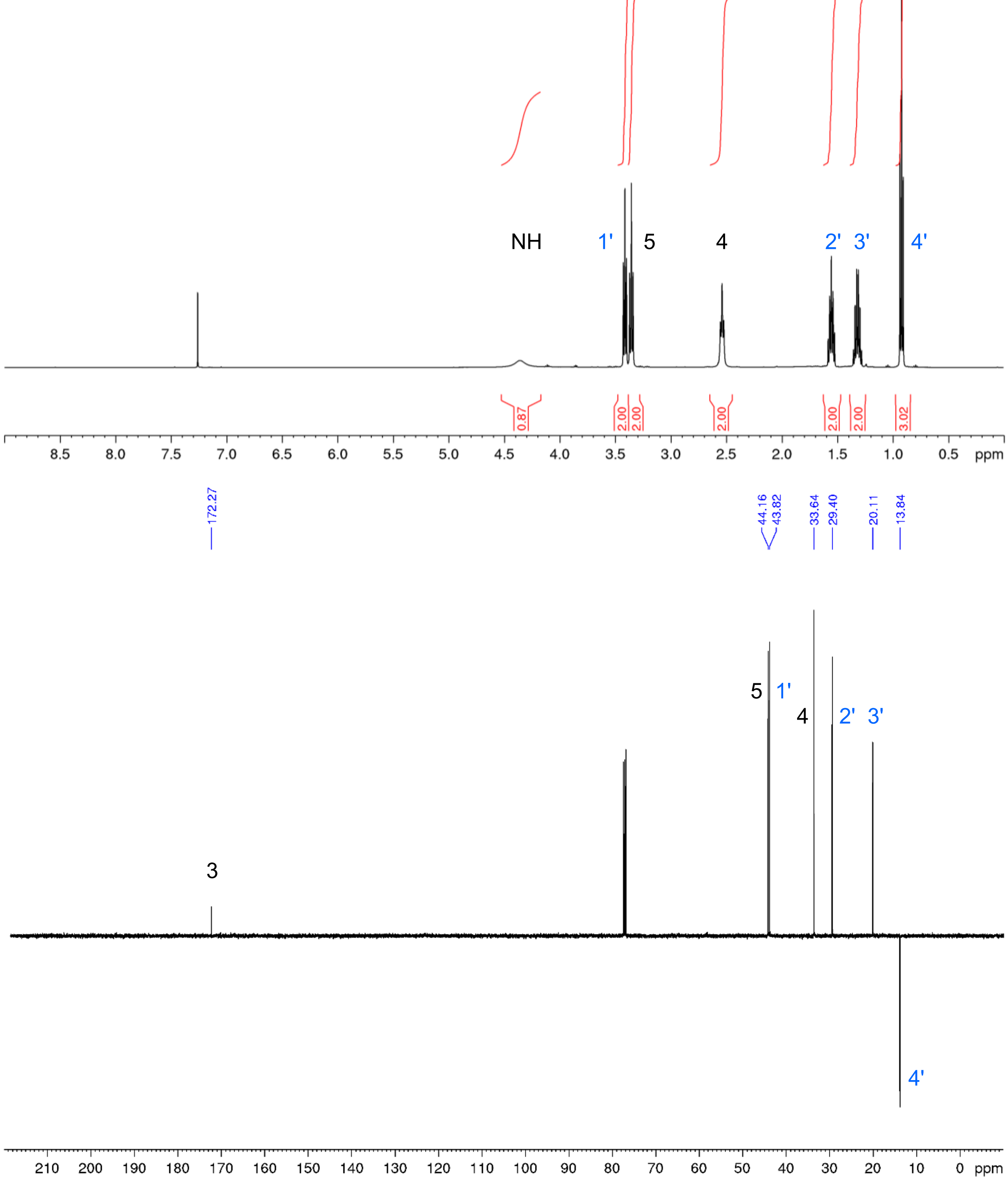

^{1}H NMR (500 MHz, $CDCl_3$) and ^{13}C Jmod NMR (125 MHz, $CDCl_3$) of **12ae**:

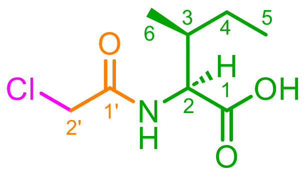

12ae

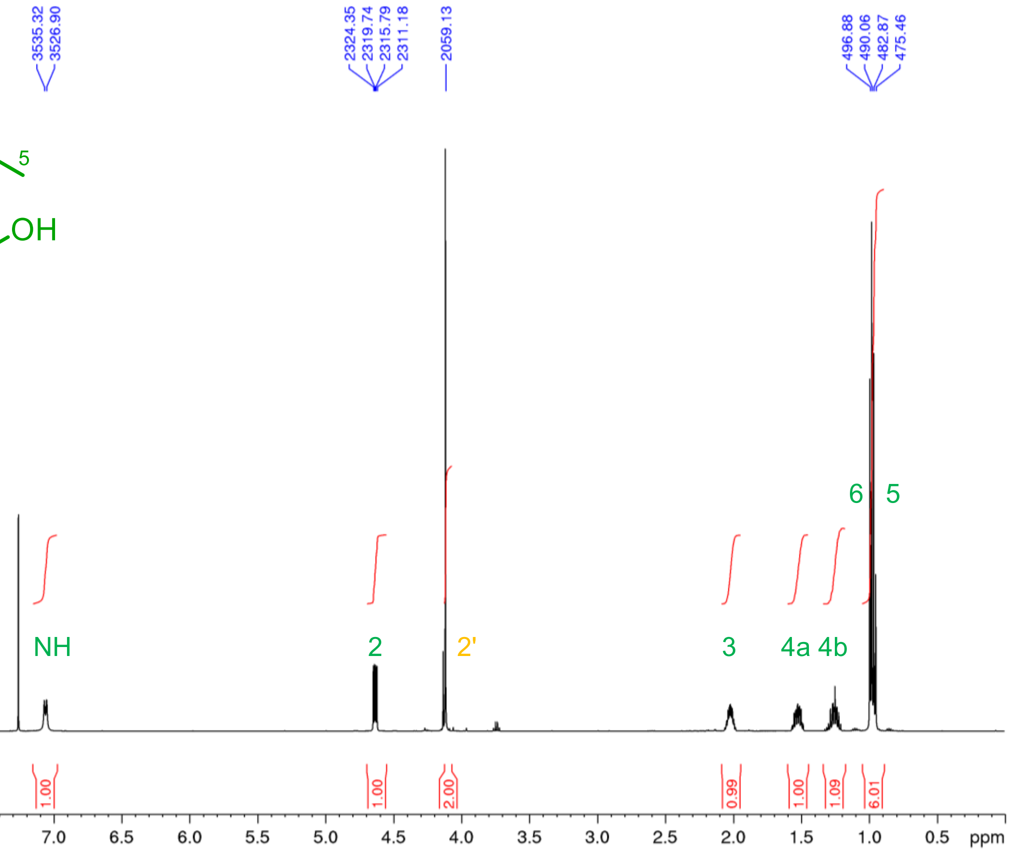

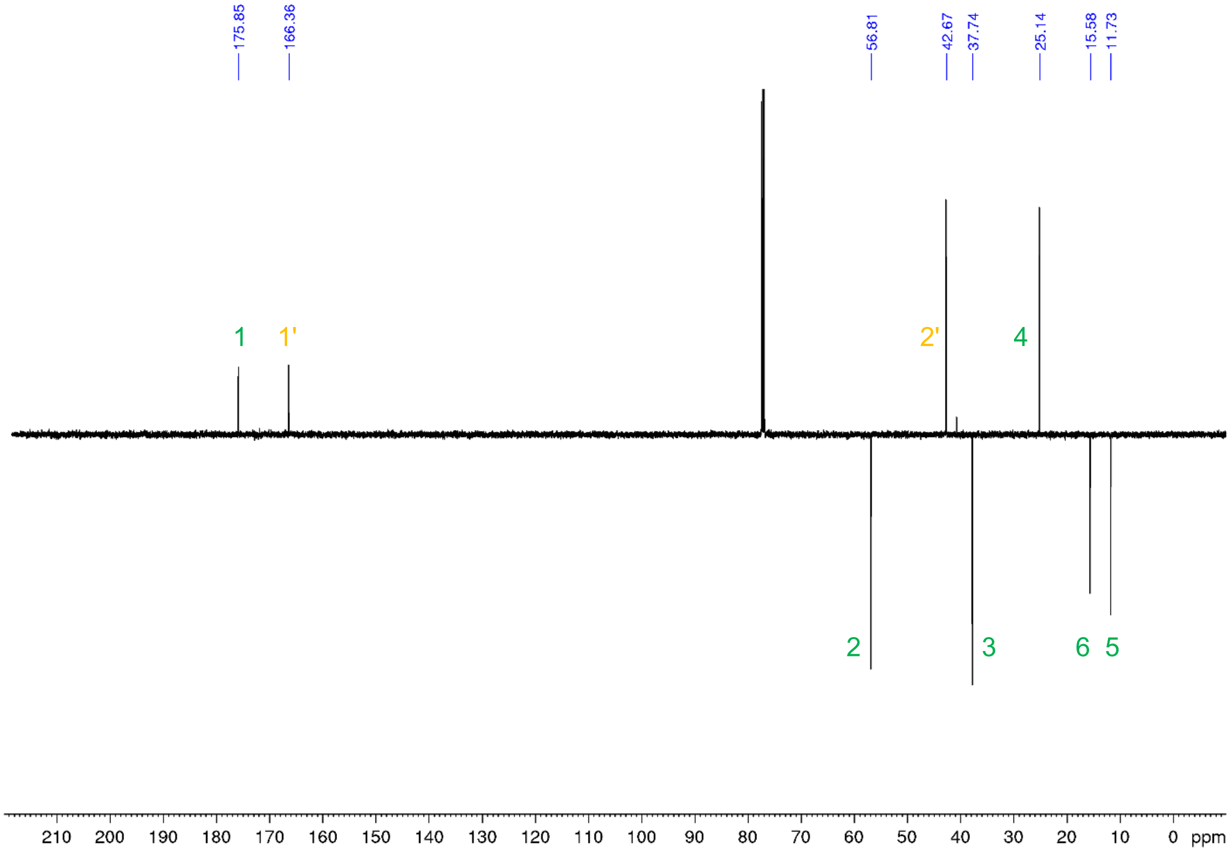

^{1}H NMR (500 MHz, $CDCl_3$) and ^{13}C Jmod NMR (125 MHz, $CDCl_3$) of **6aa**:

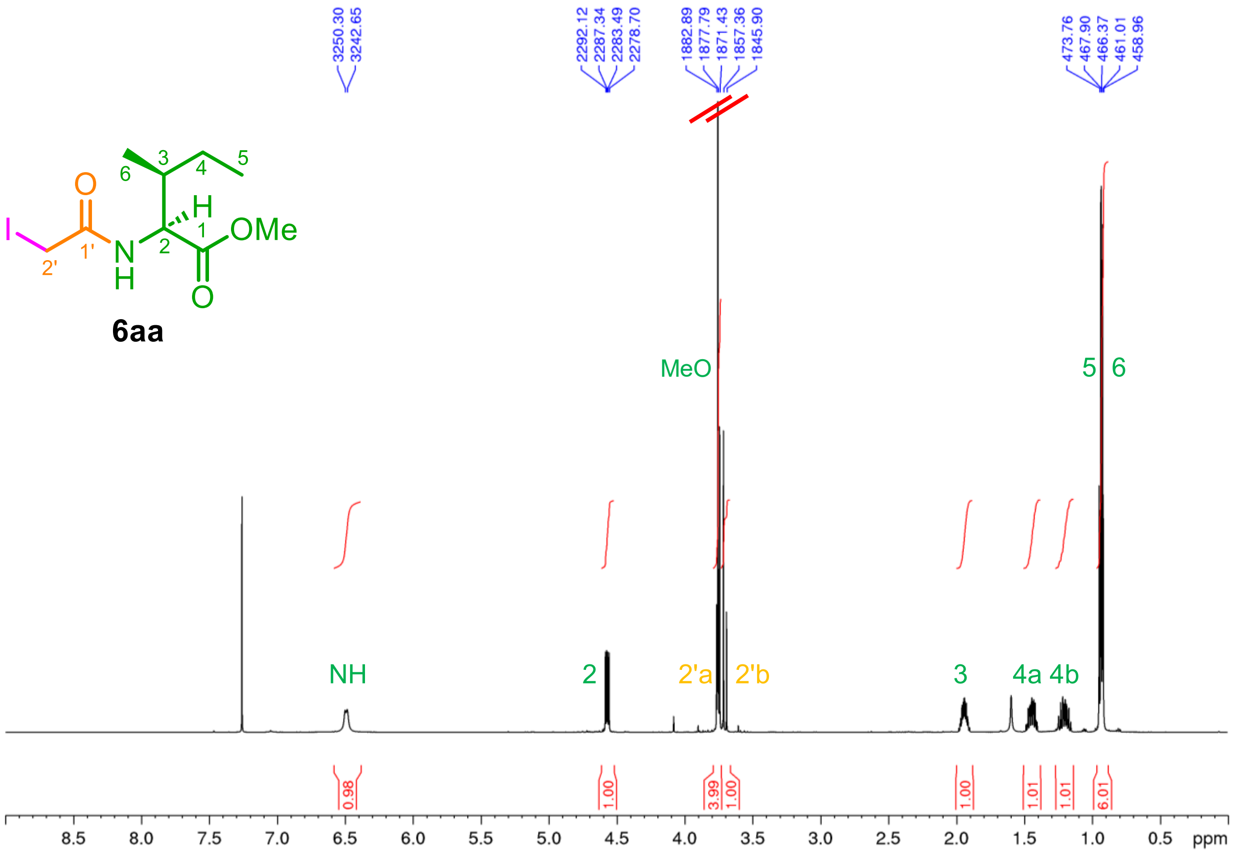

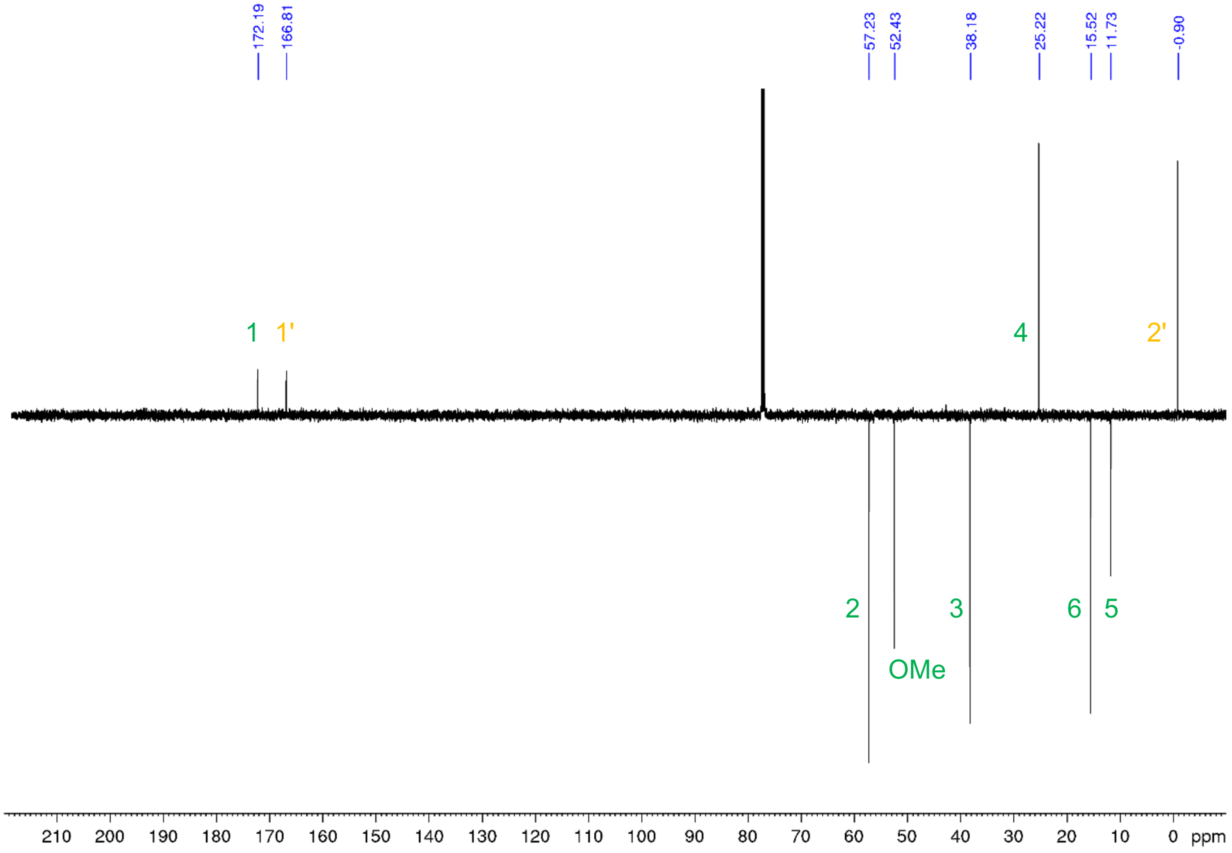

^{1}H NMR (500 MHz, $CDCl_3$) and ^{13}C Jmod NMR (125 MHz, $CDCl_3$) of **6ab**:

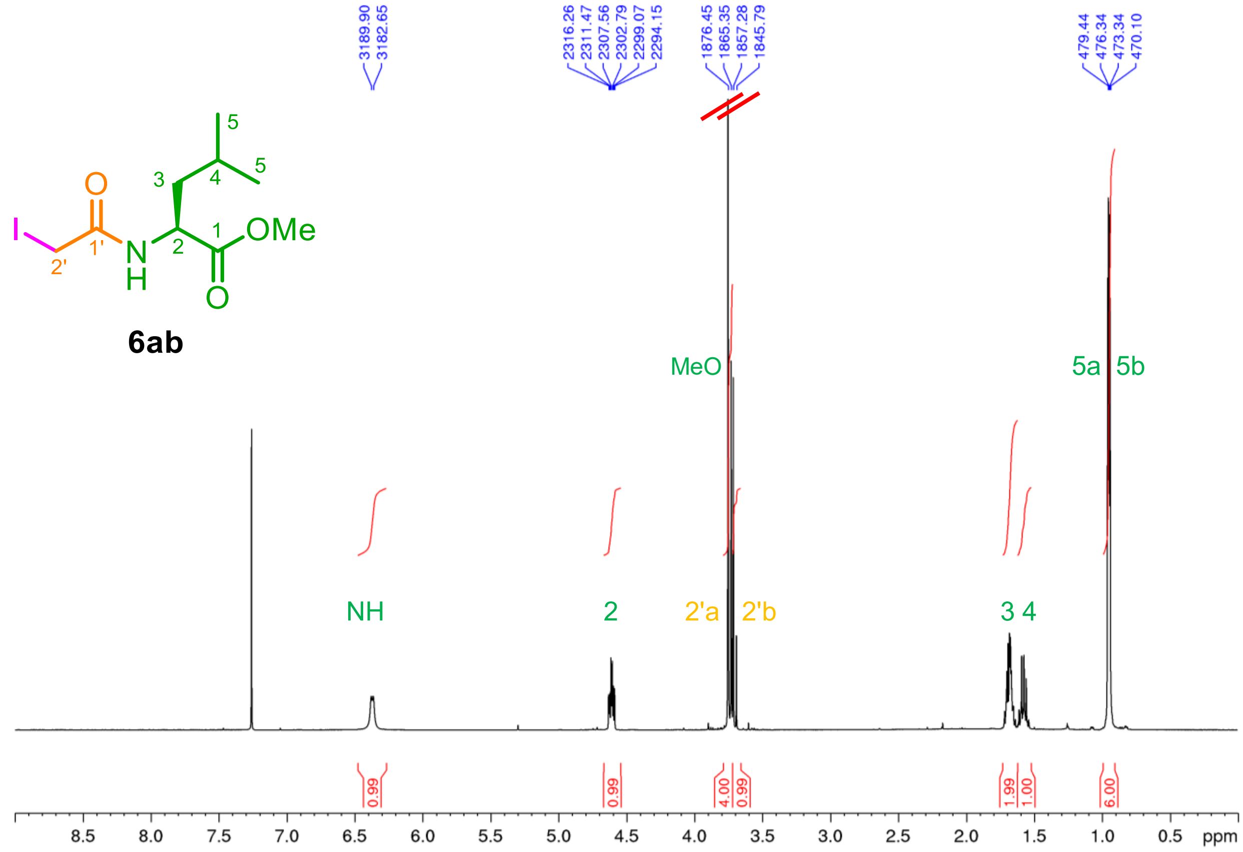

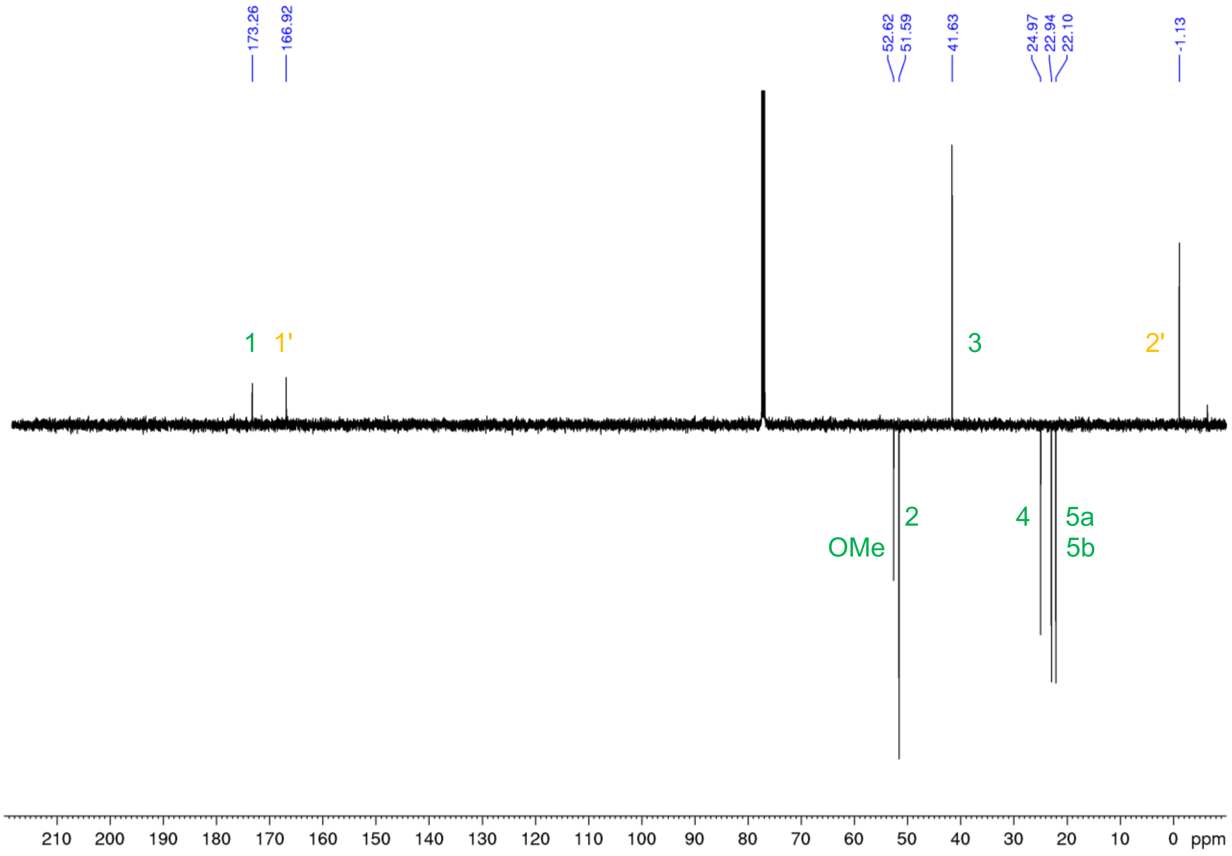

^{1}H NMR (500 MHz, $CDCl_3$) and ^{13}C Jmod NMR (125 MHz, $CDCl_3$) of **6ac**:

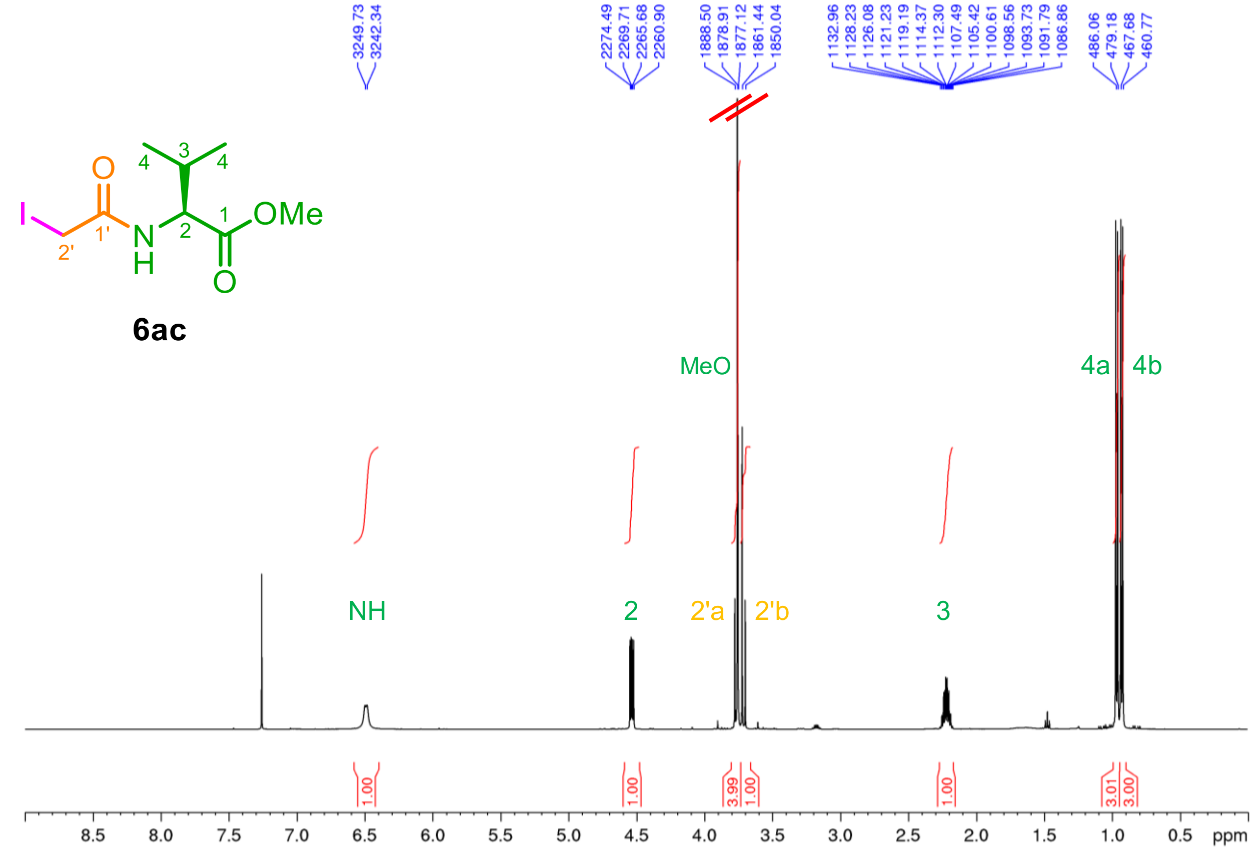

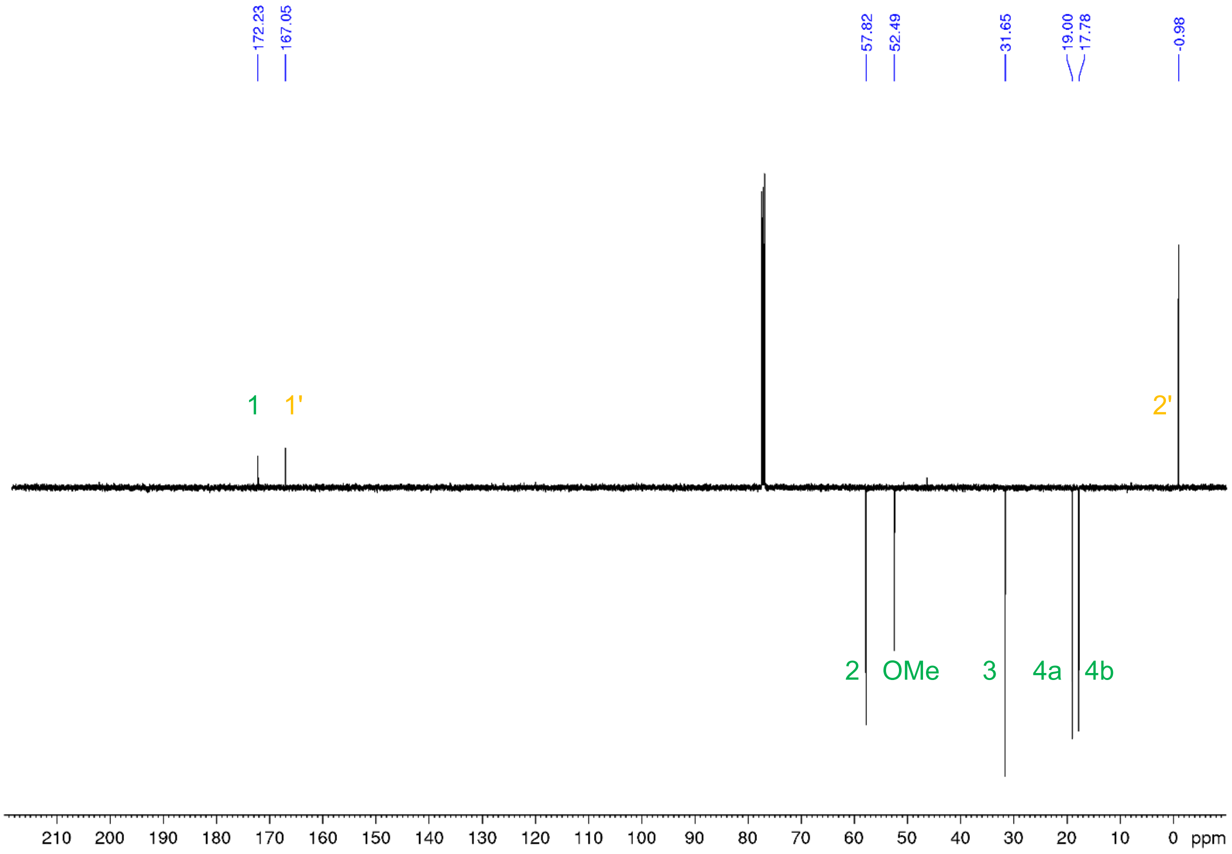

^{1}H NMR (500 MHz, $CDCl_3$) and ^{13}C Jmod NMR (125 MHz, $CDCl_3$) of **6ad**:

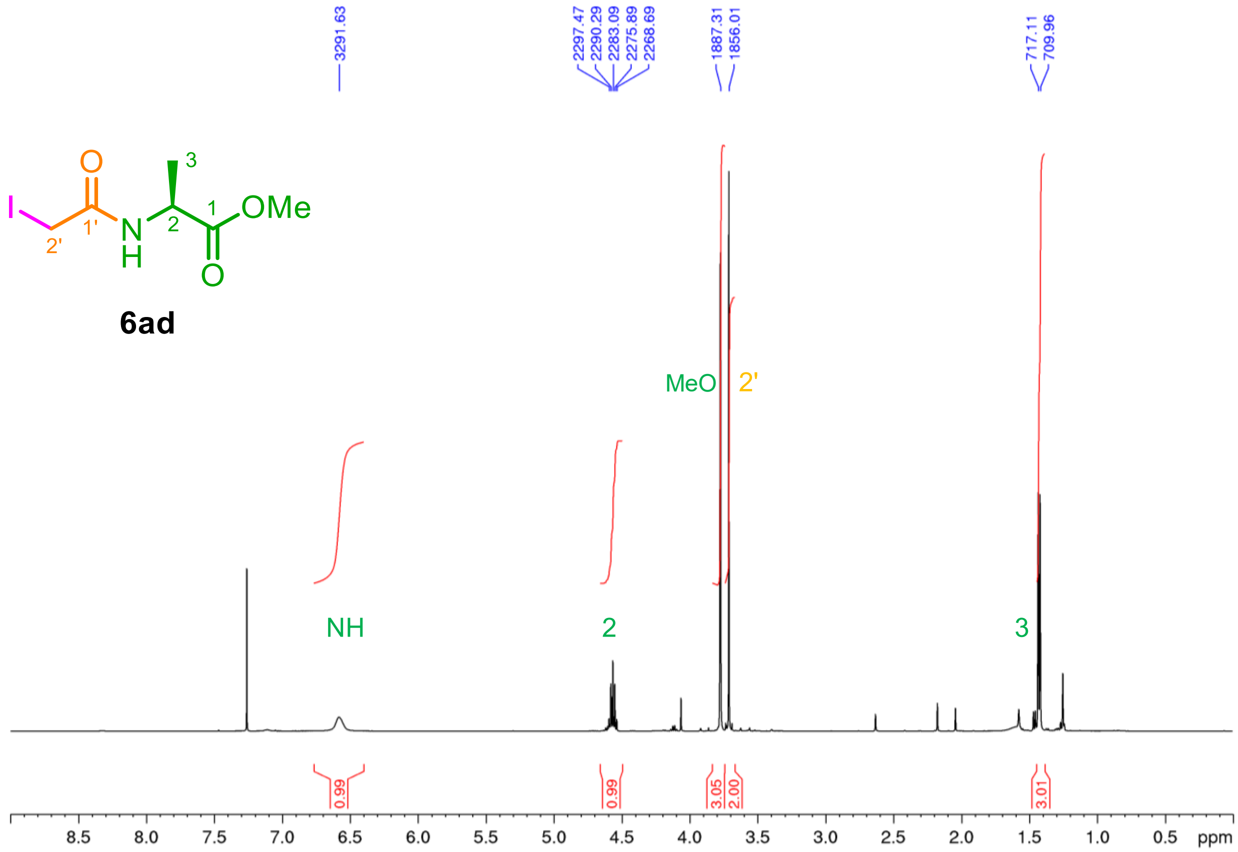

6ad

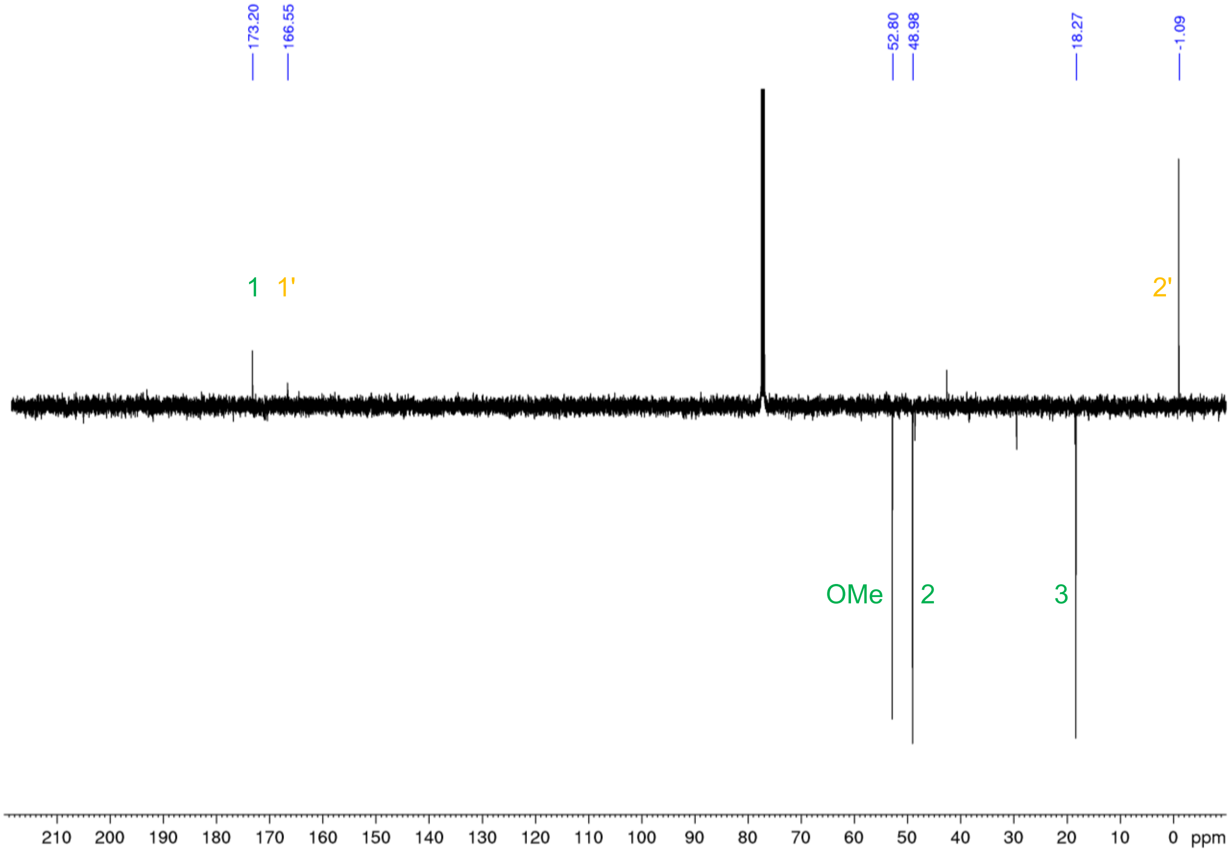

^{1}H NMR (500 MHz, CD_3OD) and ^{13}C Jmod NMR (125 MHz, CD_3OD) of **6ae**:

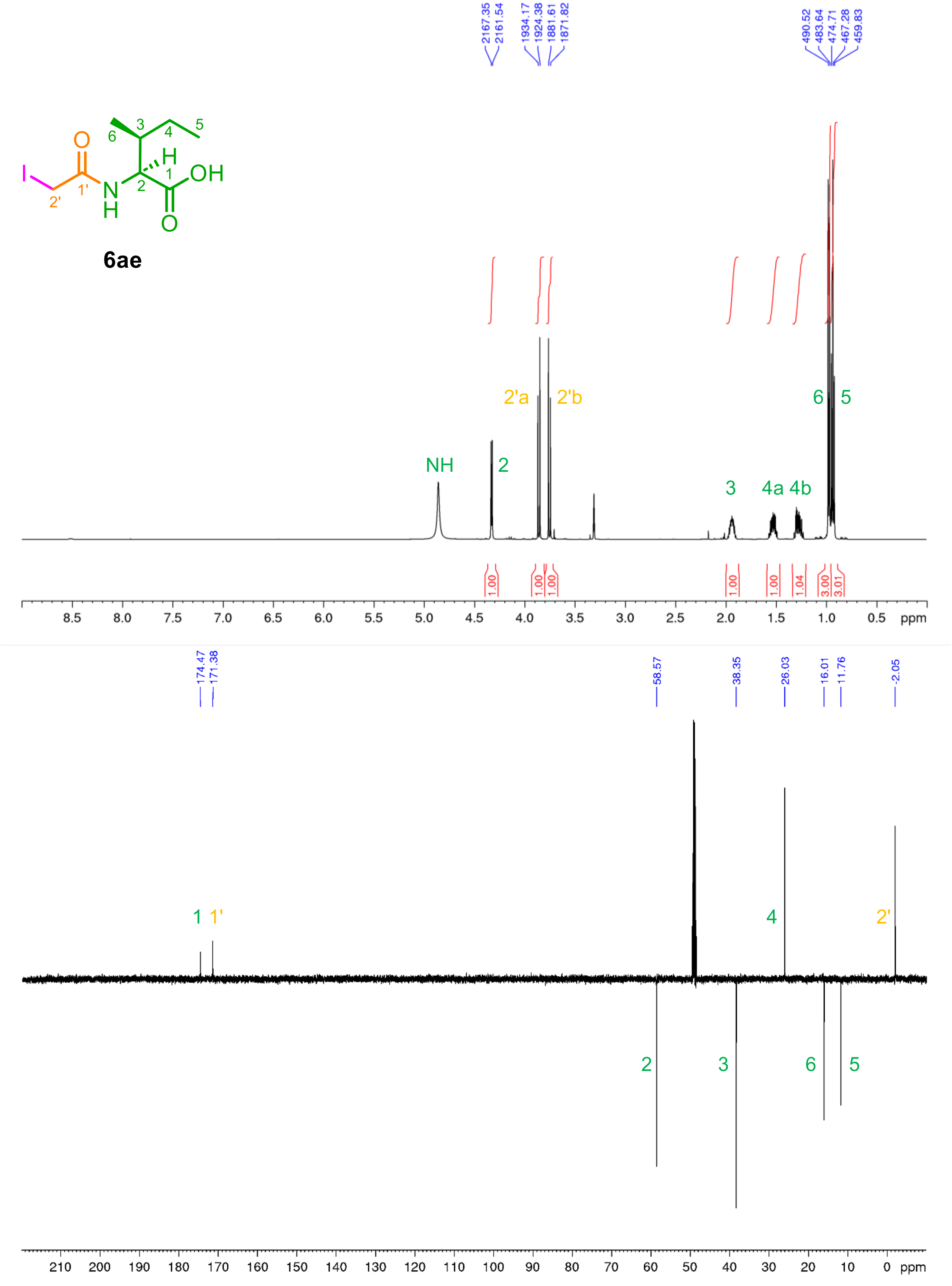

^{1}H NMR (500 MHz, $CDCl_3$) and ^{13}C Jmod NMR (125 MHz, $CDCl_3$) of **6ba**:

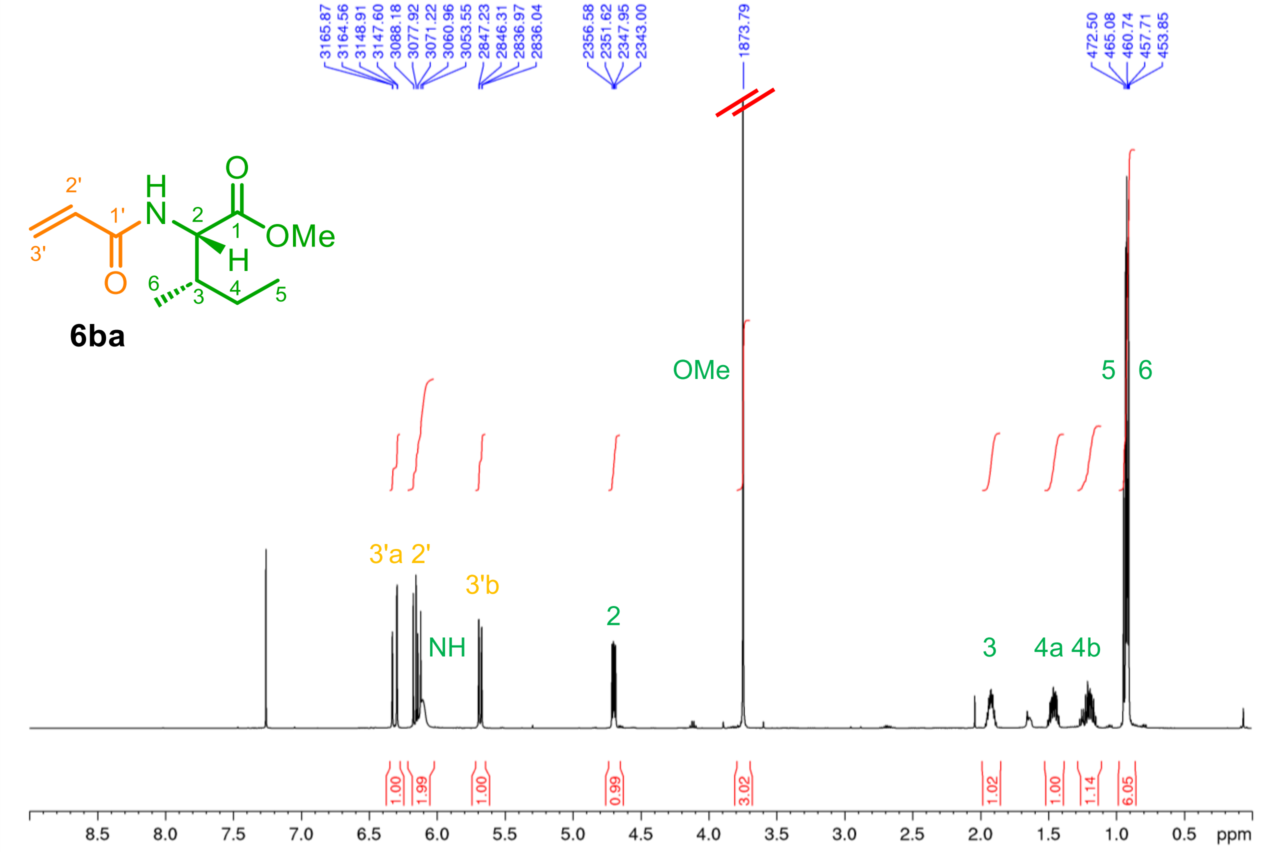

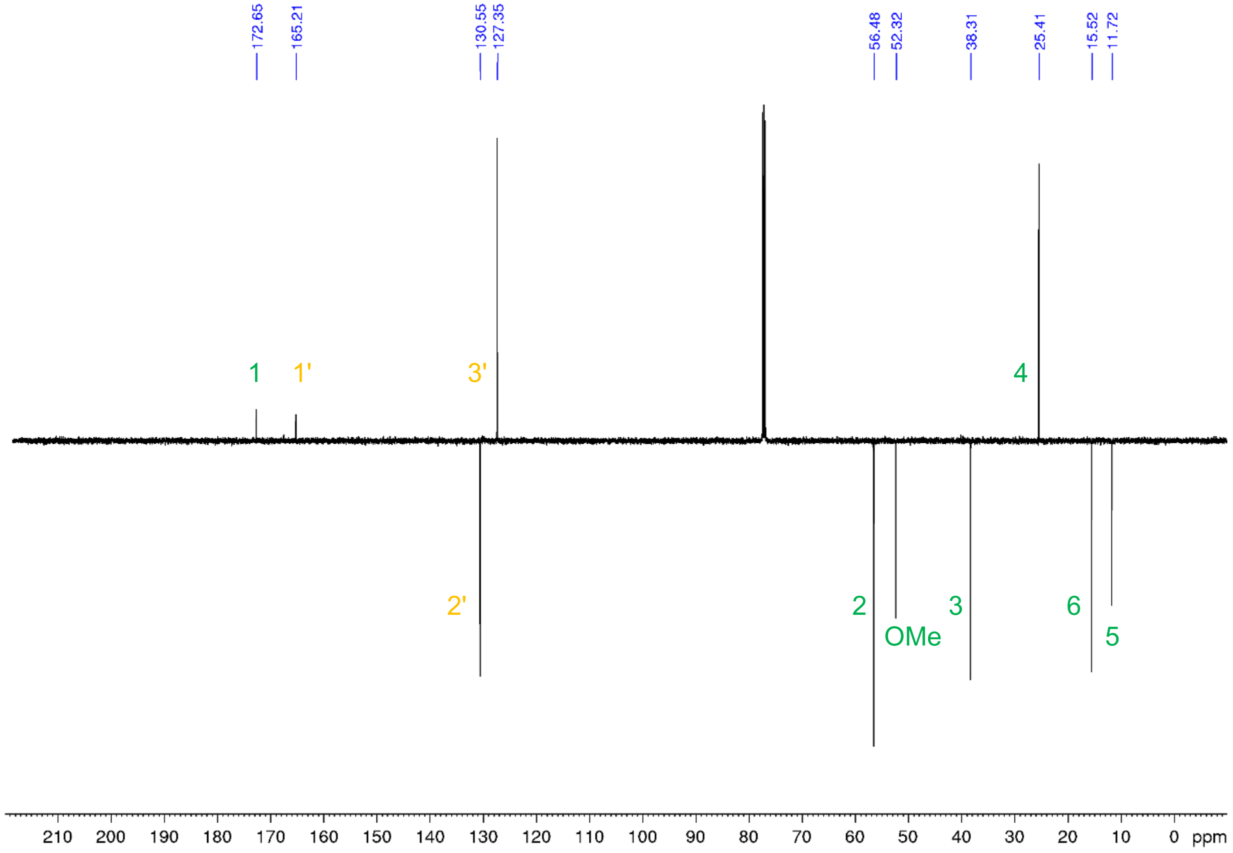

^{1}H NMR (500 MHz, $CDCl_3$) and ^{13}C Jmod NMR (125 MHz, $CDCl_3$) of **3aaa**:

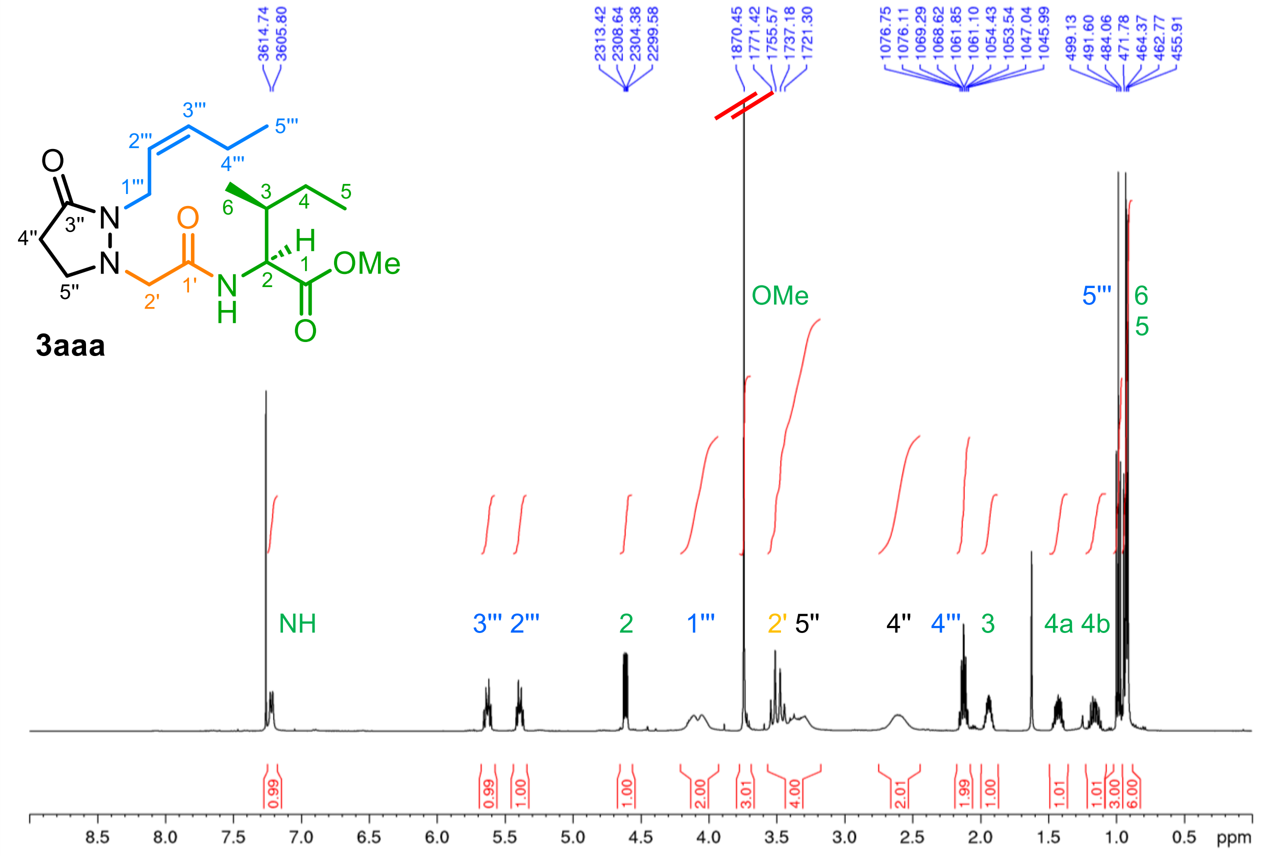

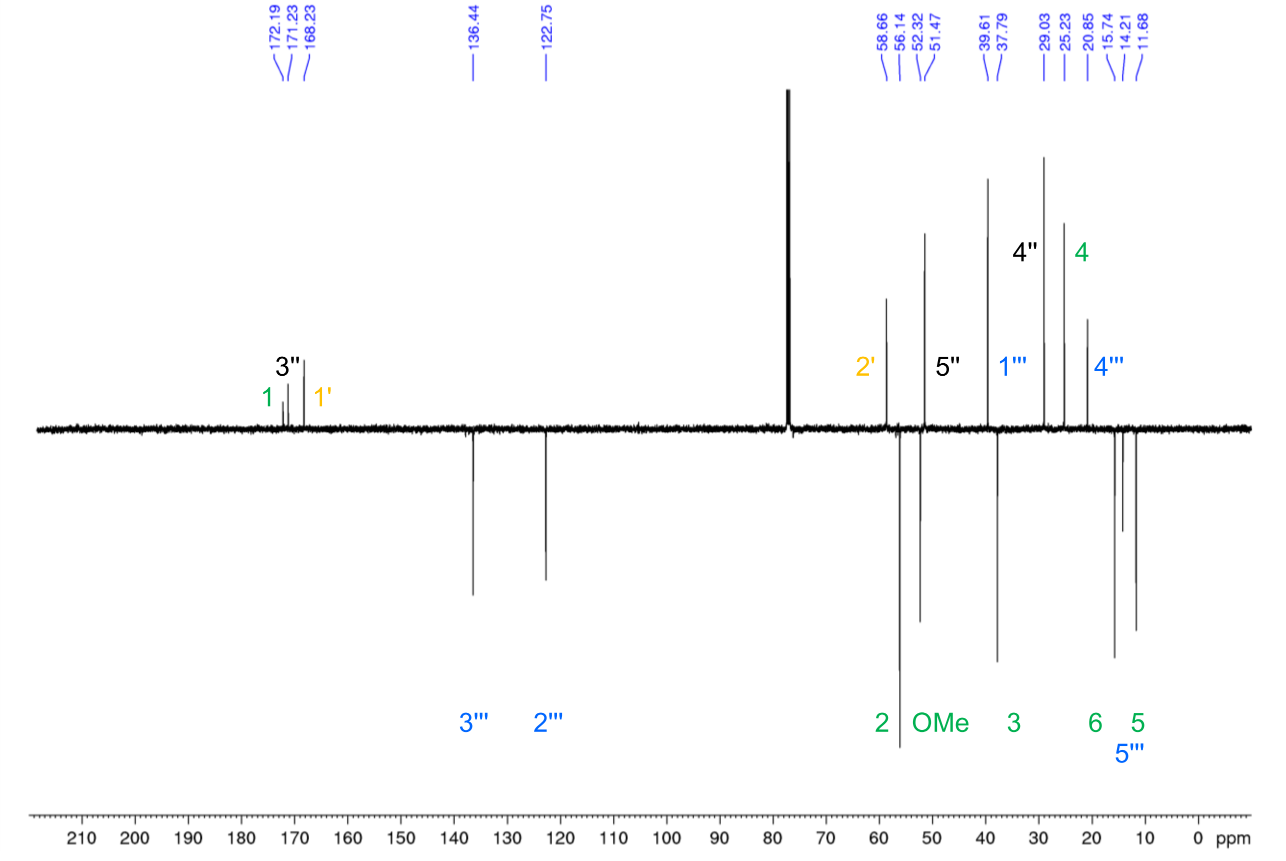

^{1}H NMR (500 MHz, $CDCl_3$) and ^{13}C Jmod NMR (125 MHz, $CDCl_3$) of **3aab**:

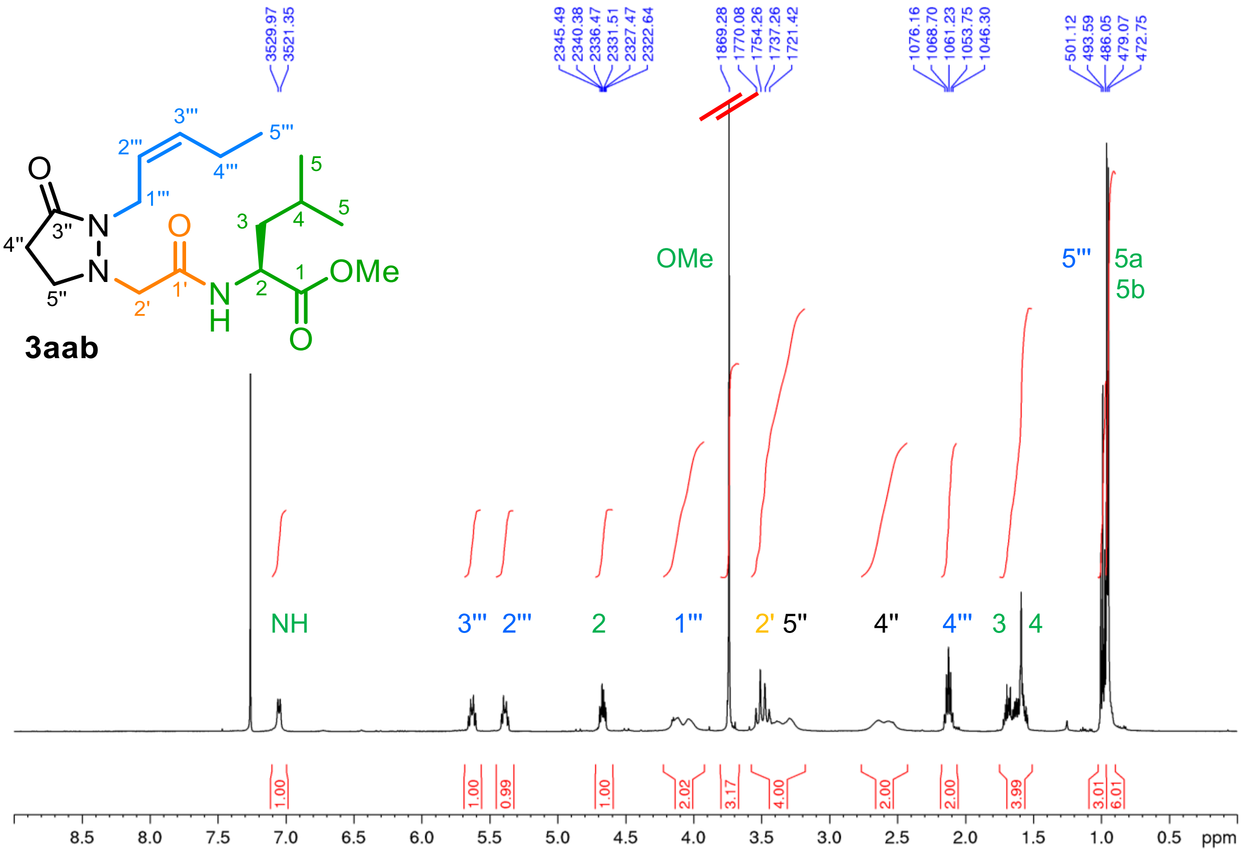

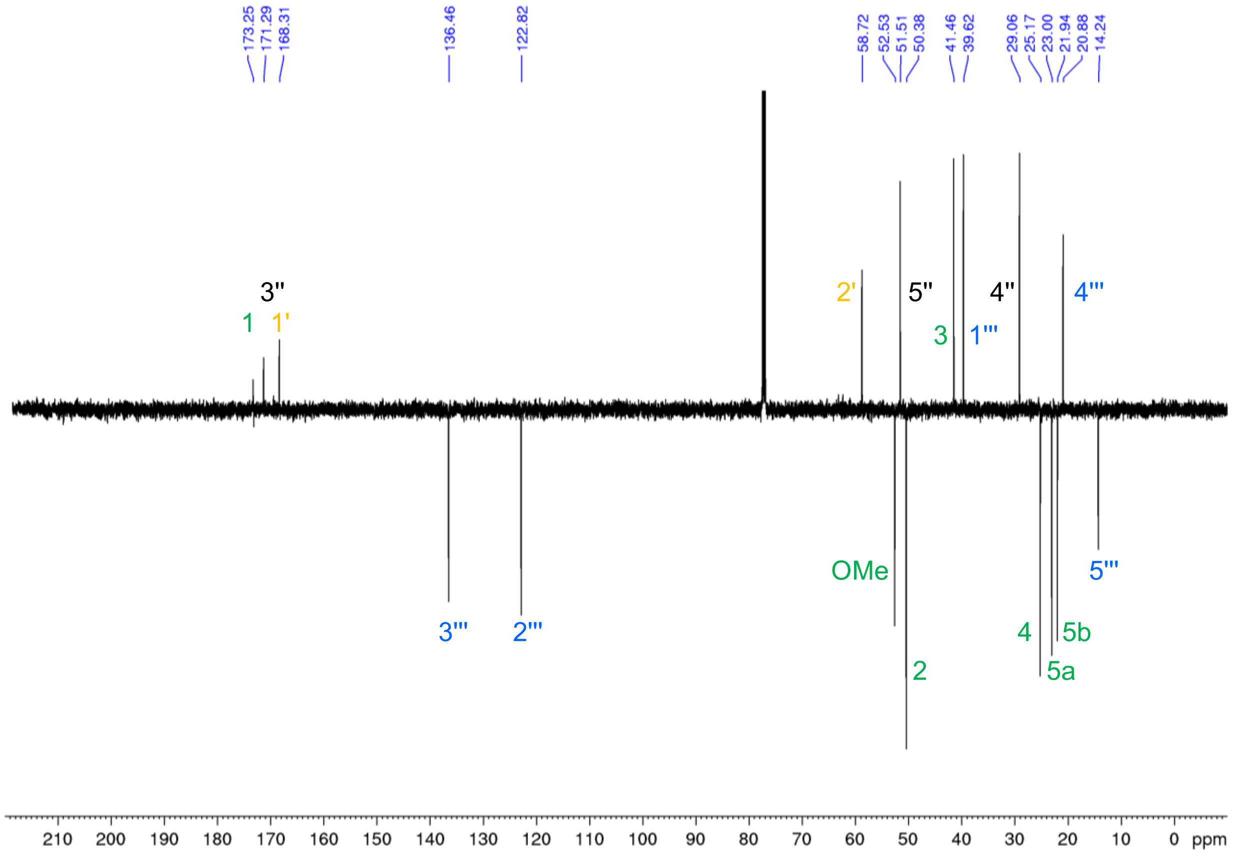

^{1}H NMR (500 MHz, $CDCl_3$) and ^{13}C Jmod NMR (125 MHz, $CDCl_3$) of **3aac**:

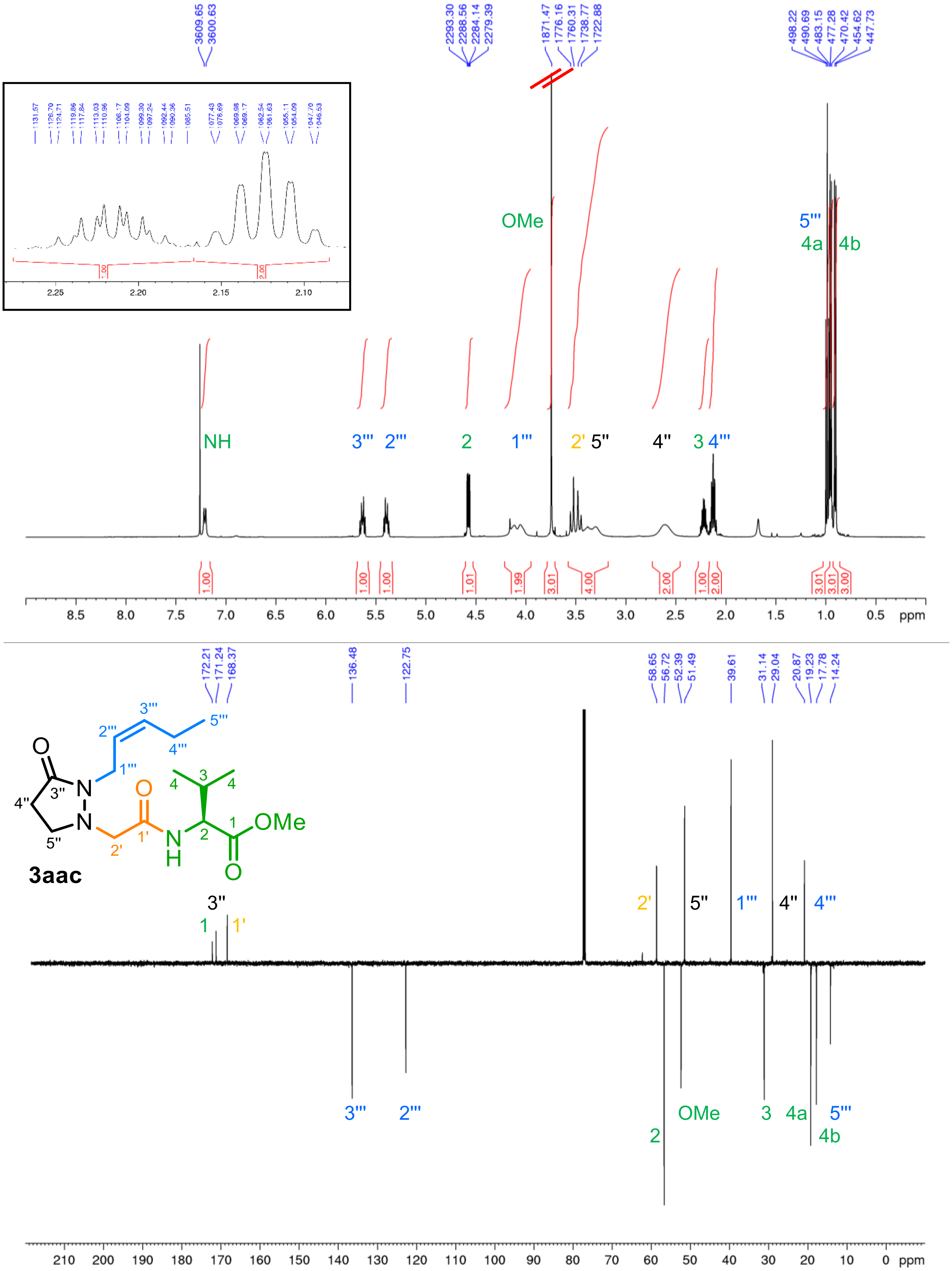

^{1}H NMR (300 MHz, $CDCl_3$) and ^{13}C Jmod NMR (75 MHz, $CDCl_3$) of **3aad**:

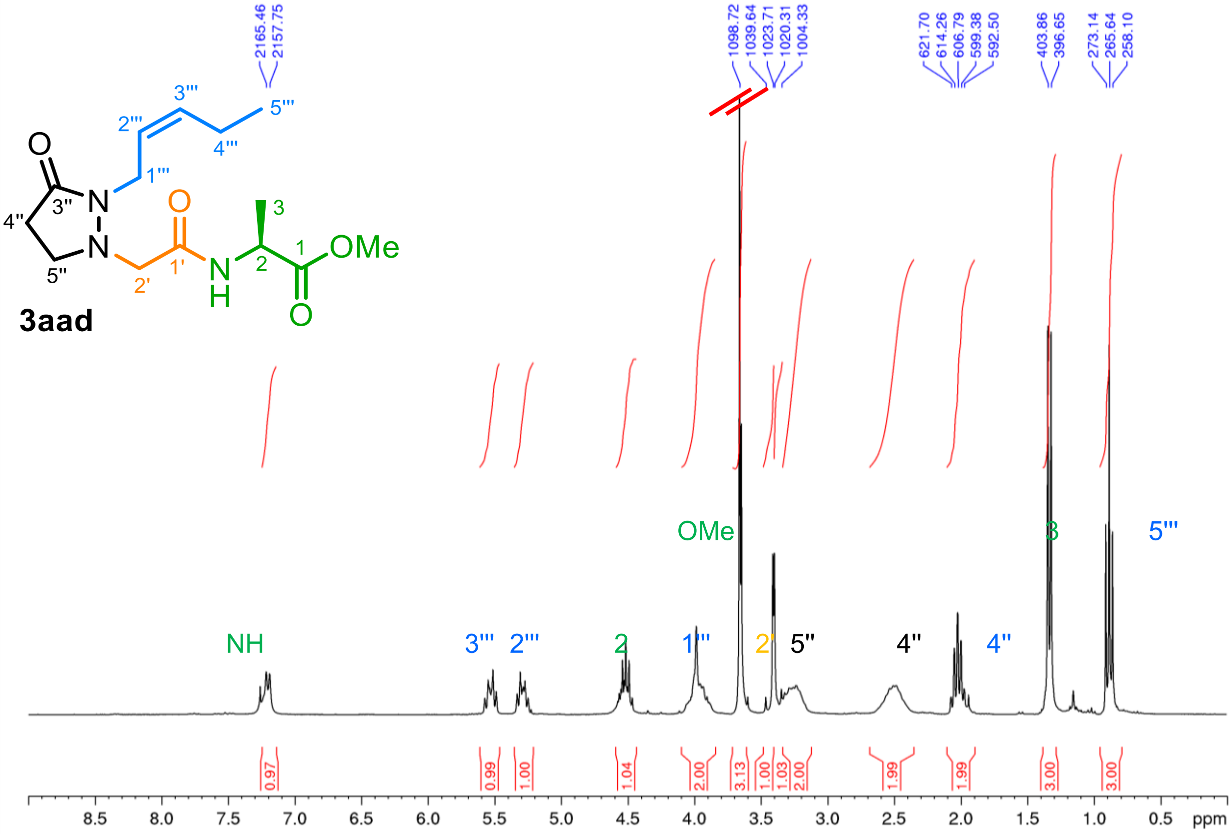

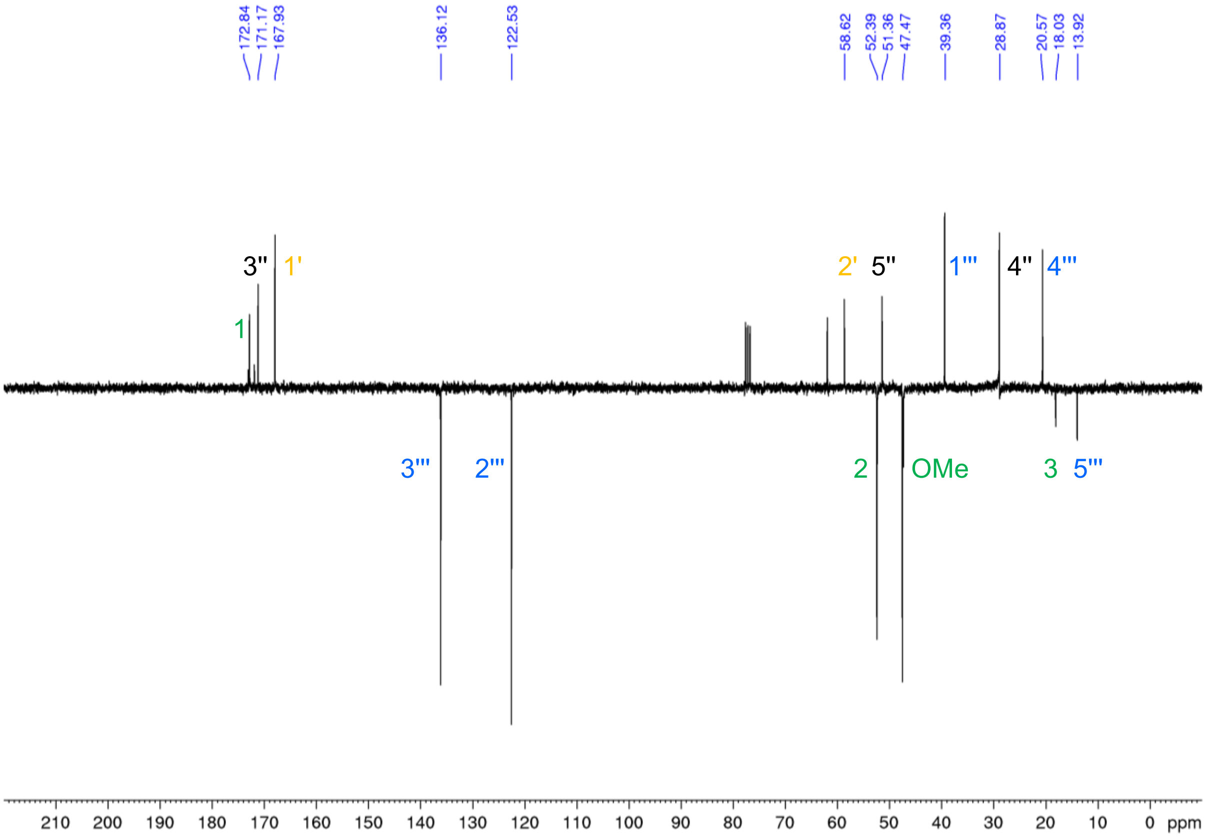

^{1}H NMR (300 MHz, $CDCl_3$) and ^{13}C Jmod NMR (125 MHz, $CDCl_3$) of **3aae**:

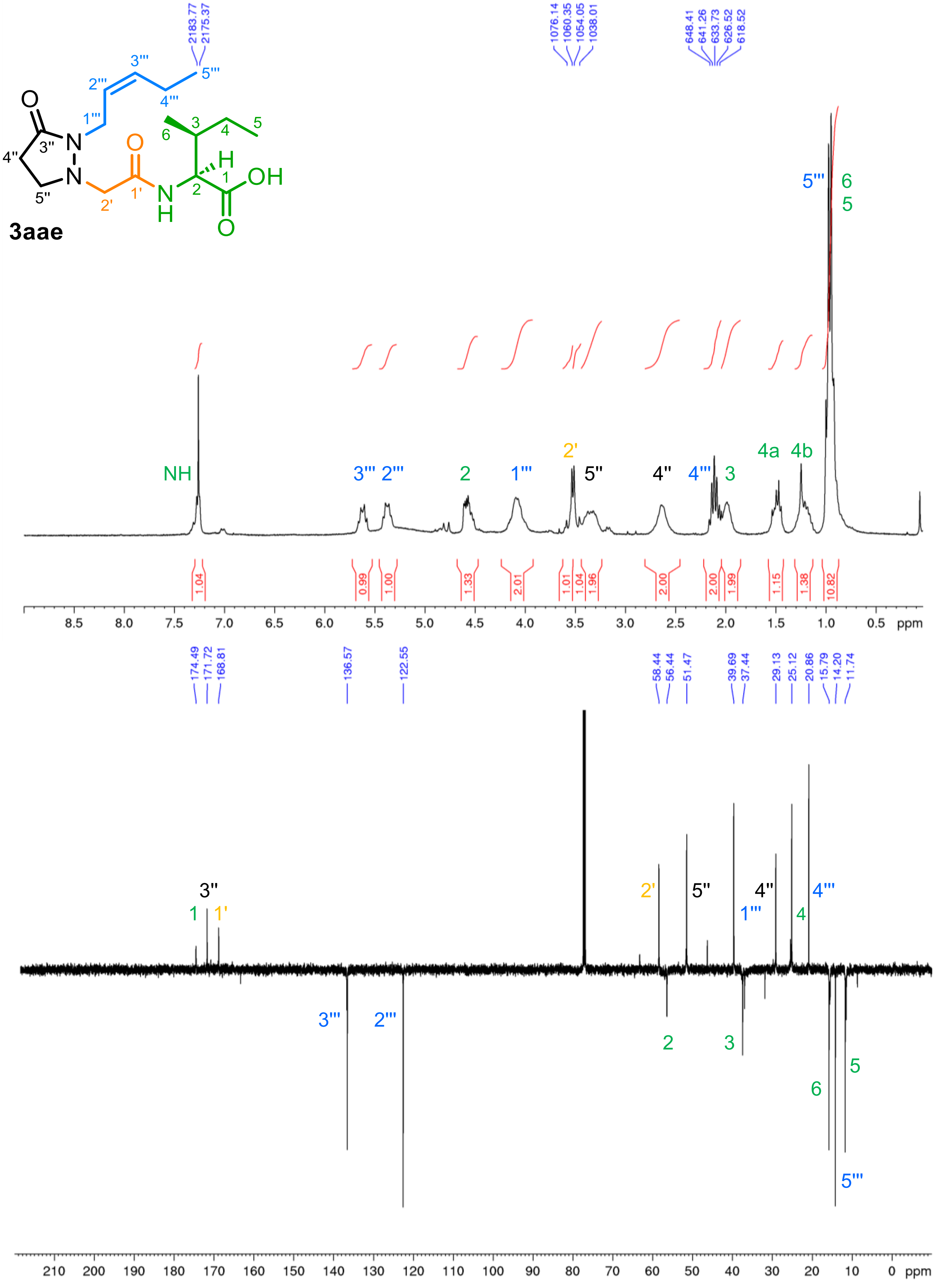

^{1}H NMR (500 MHz, $CDCl_3$) and ^{13}C Jmod NMR (125 MHz, $CDCl_3$) of **3baa**:

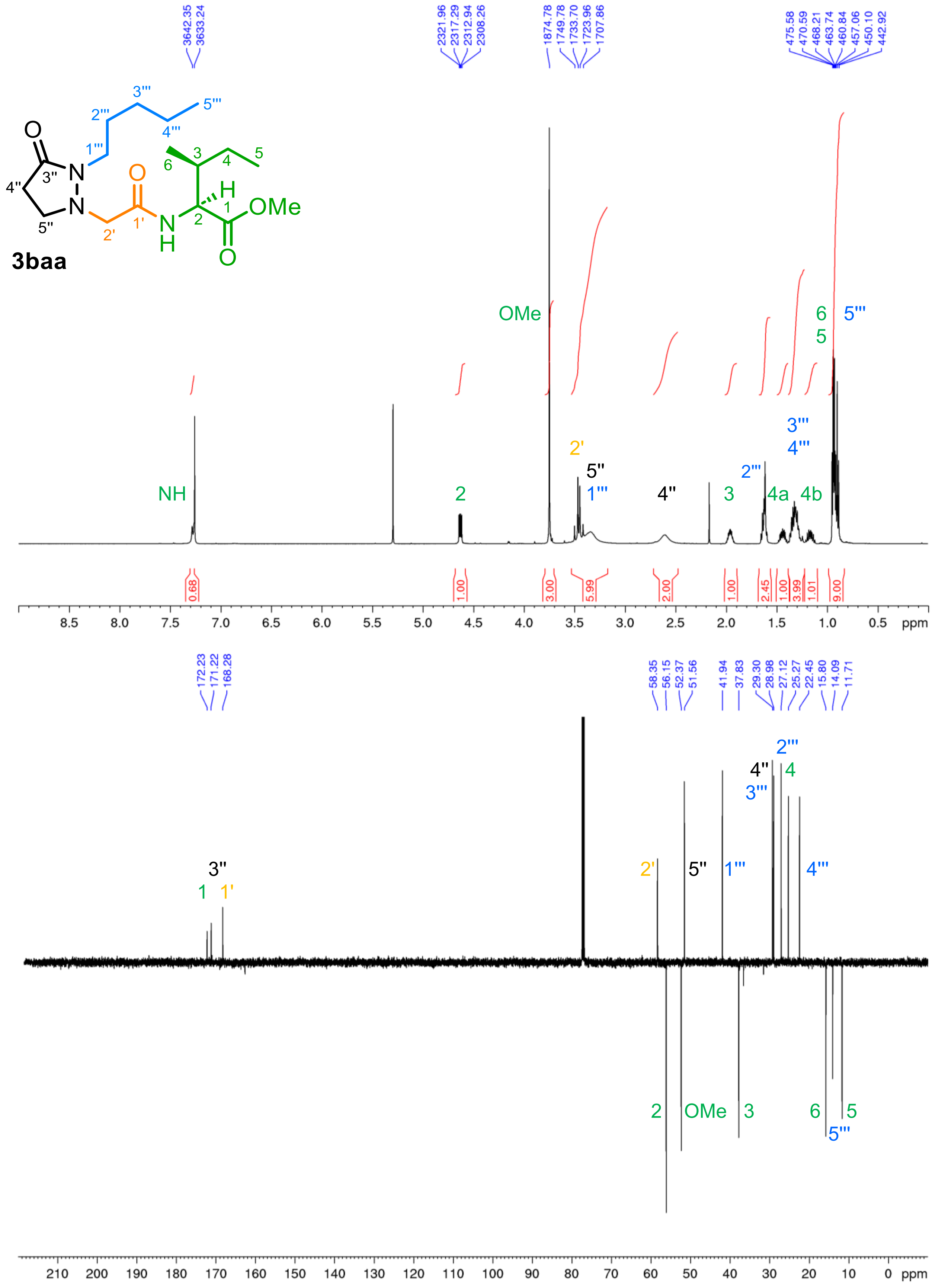

^{1}H NMR (500 MHz, $CDCl_3$) and ^{13}C Jmod NMR (125 MHz, $CDCl_3$) of **3caa**:

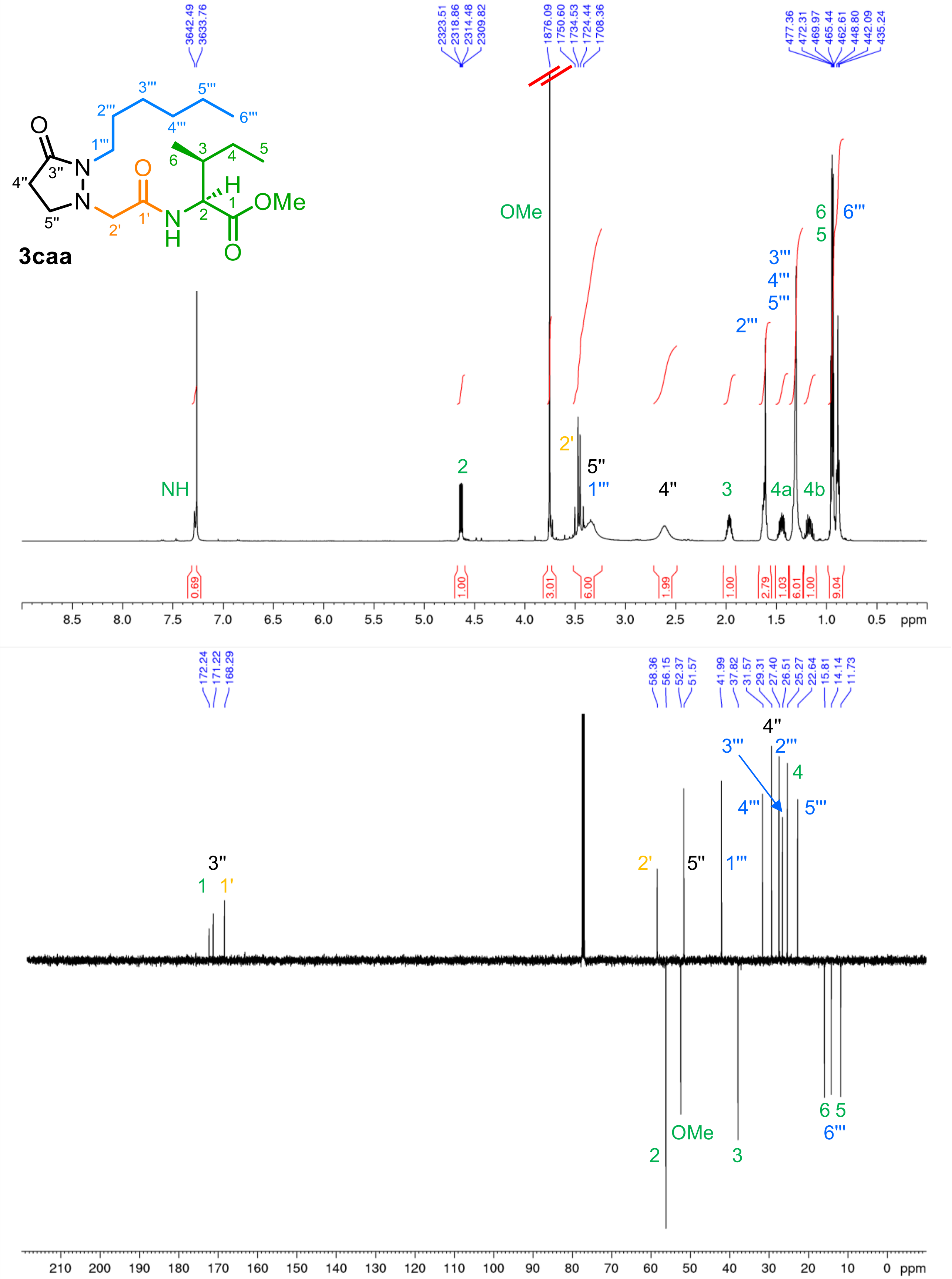

^{1}H NMR (500 MHz, $CDCl_3$) and ^{13}C Jmod NMR (125 MHz, $CDCl_3$) of **3daa**:

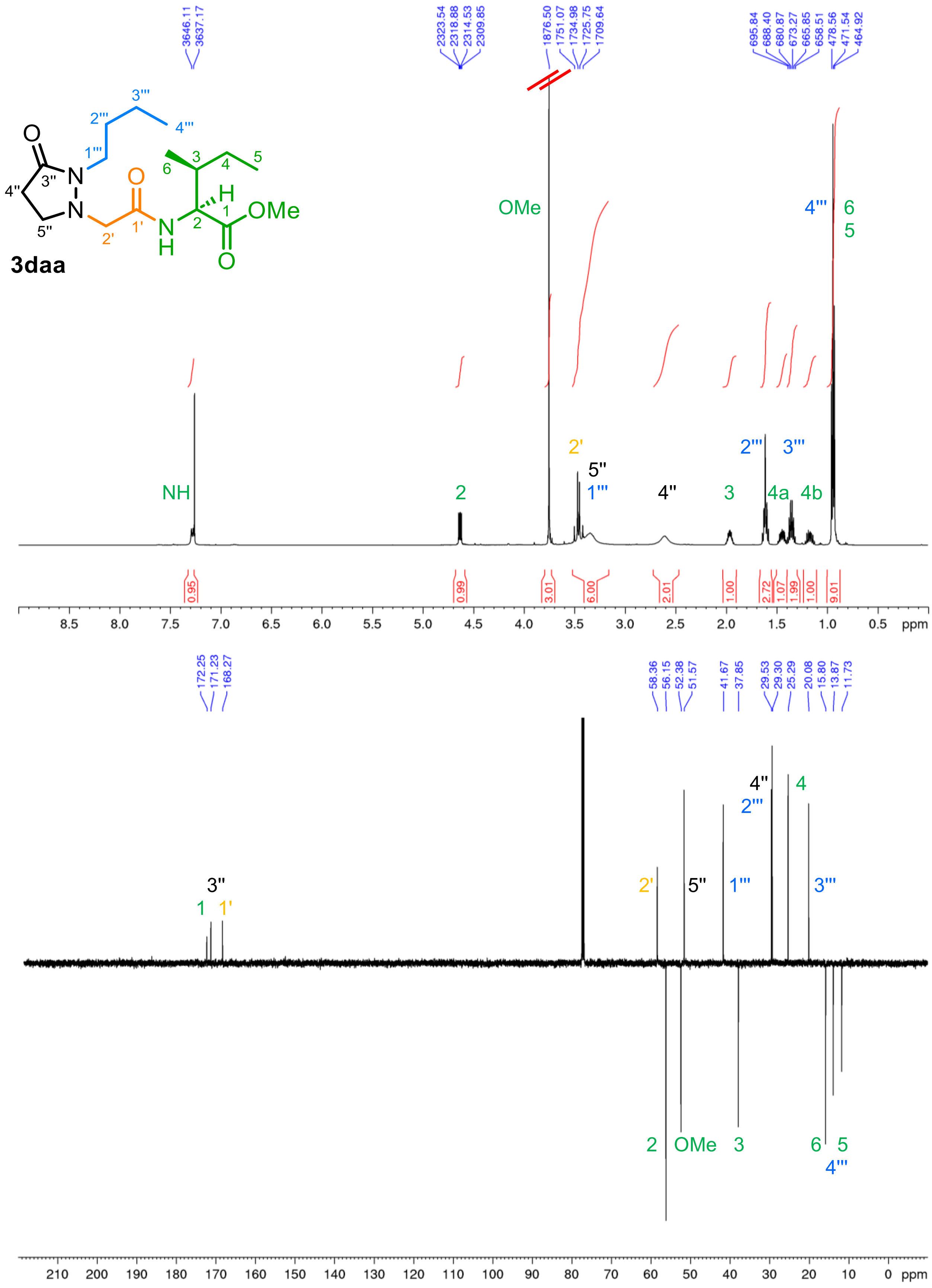

^{1}H NMR (500 MHz, $CDCl_3$) and ^{13}C Jmod NMR (125 MHz, $CDCl_3$) of **3aba**:

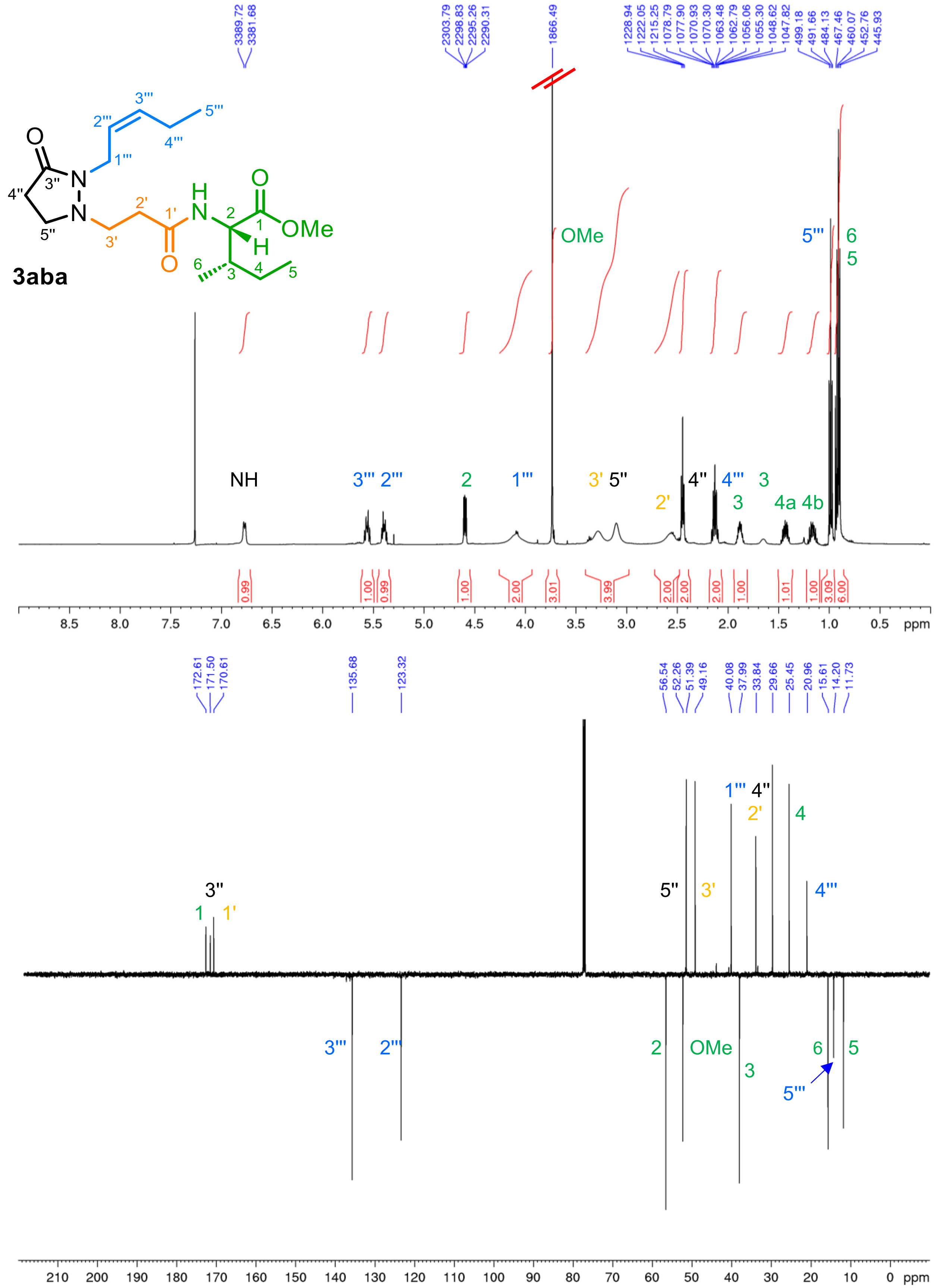

^{1}H NMR (500 MHz, $CDCl_3$) and ^{13}C Jmod NMR (125 MHz, $CDCl_3$) of **3bab**:

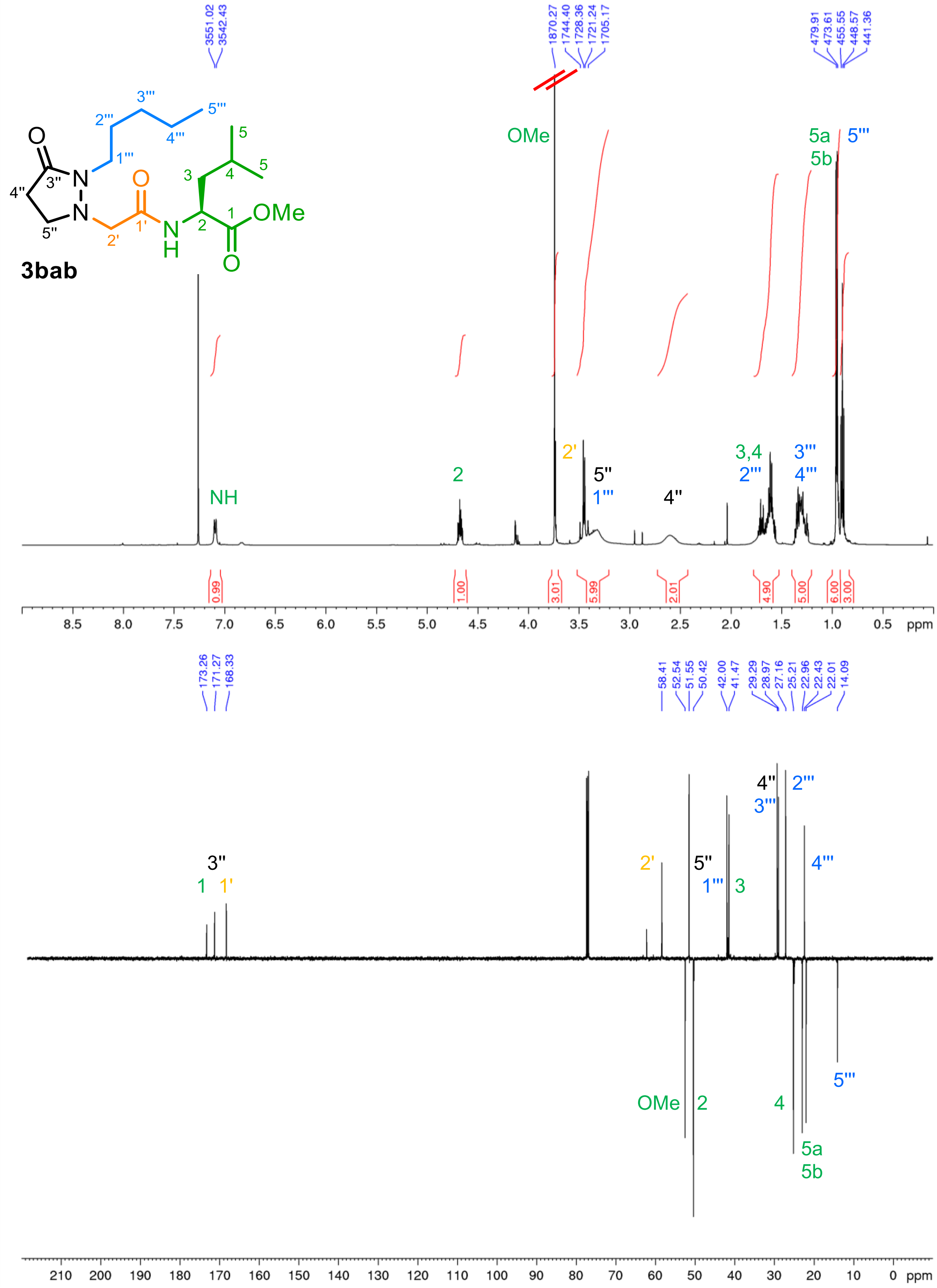

^{1}H NMR (300 MHz, $CDCl_3$) and ^{13}C Jmod NMR (75 MHz, $CDCl_3$) of **3bac**:

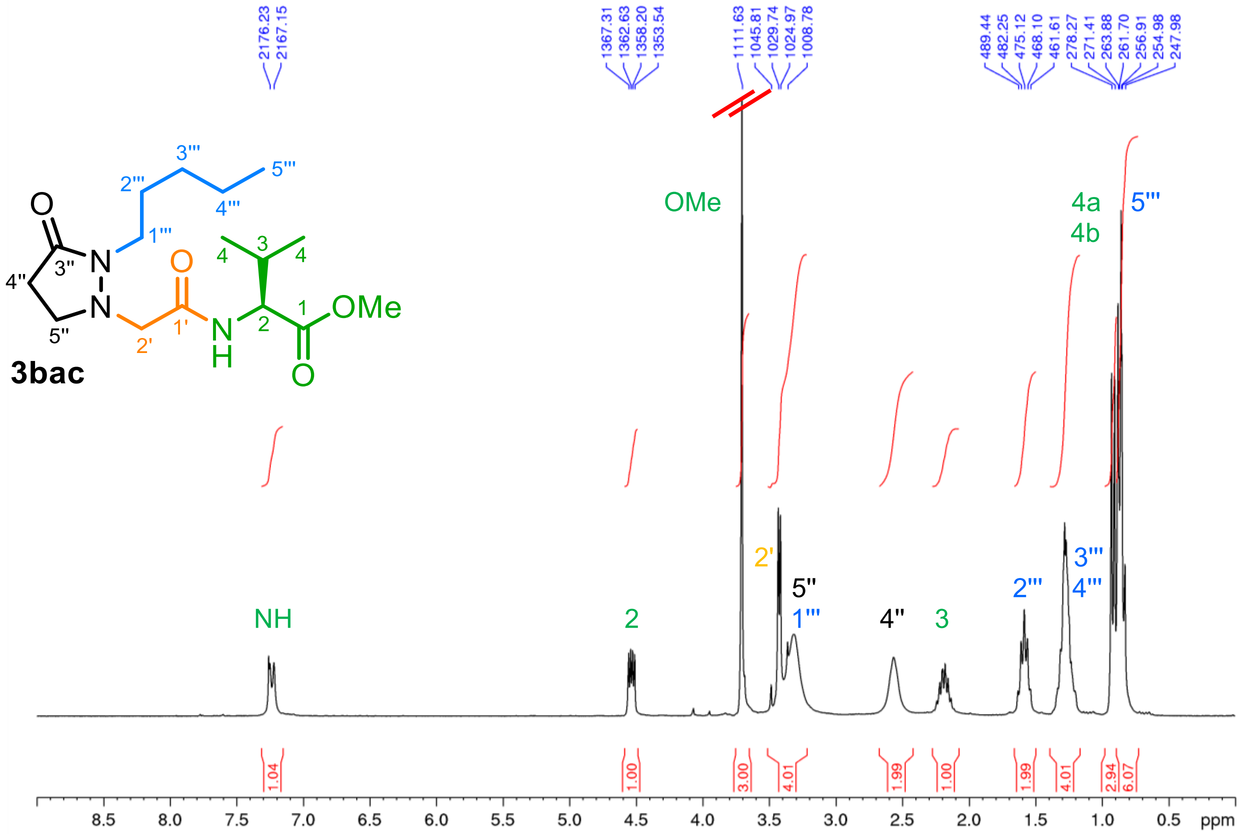

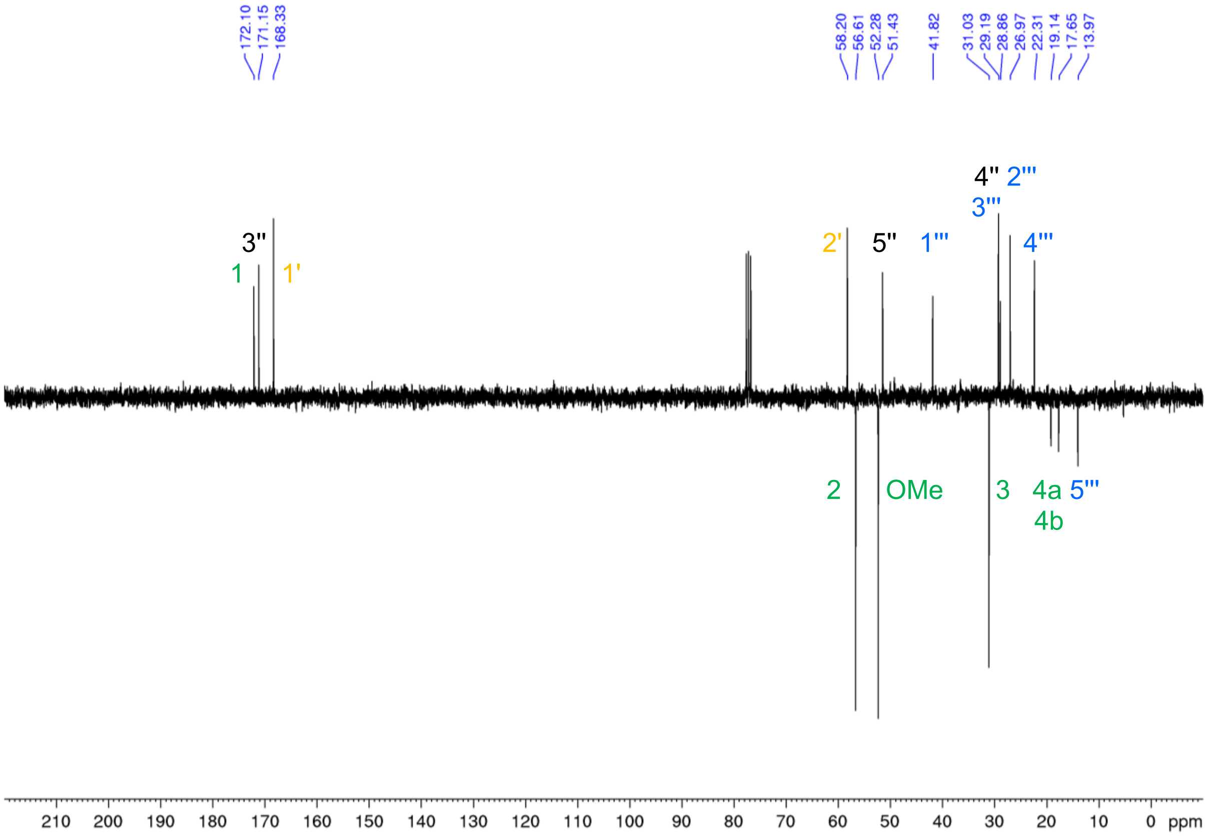

Supplement: RA-014-D3RA07887F-s001 [file RA-014-D3RA07887F-s001.pdf]
